# Asymmetric Total Synthesis and Structural Reassignment of Nervione

**DOI:** 10.1021/acs.joc.5c00258

**Published:** 2025-03-19

**Authors:** Kunita Phakdeeyothin, Jian-Liang Li, Yi-Tian Hong, Rong-Jie Chein

**Affiliations:** † Institute of Chemistry, Academia Sinica, Taipei 11529, Taiwan; ‡ Department of Chemistry, National Taiwan University, Taipei 10617, Taiwan; § Department of Chemistry, National Taiwan Normal University, Taipei 11677, Taiwan

## Abstract

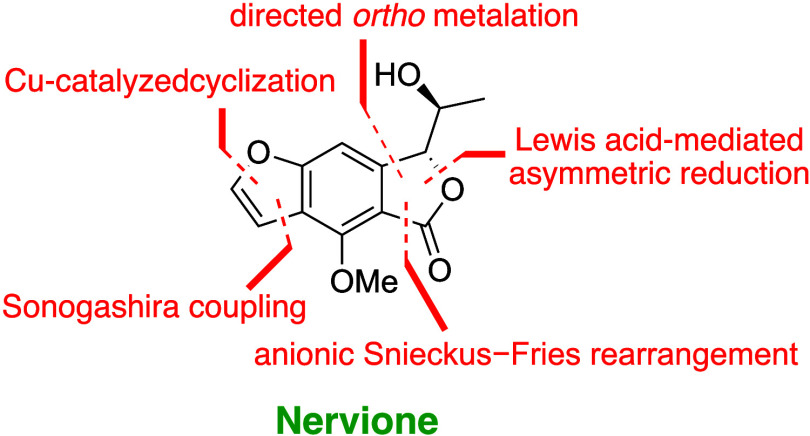

We report the asymmetric total synthesis of optically active nervione in both (+) and (−) forms, a natural product initially isolated from *Nervilia concolor* in 2022. Beginning with commercially available resorcinol, optically pure nervione was synthesized in 10 steps, employing a combination of *ortho*-directed strategies and a crucial late-stage, Lewis acid-mediated, highly diastereoselective reduction. Discrepancies observed in circular dichroism (CD) spectra prompted the reassignment of nervione’s absolute configuration from (3*S*, 8*R*) to (3*R*, 8*S*).

## Introduction

**Figure 1:**
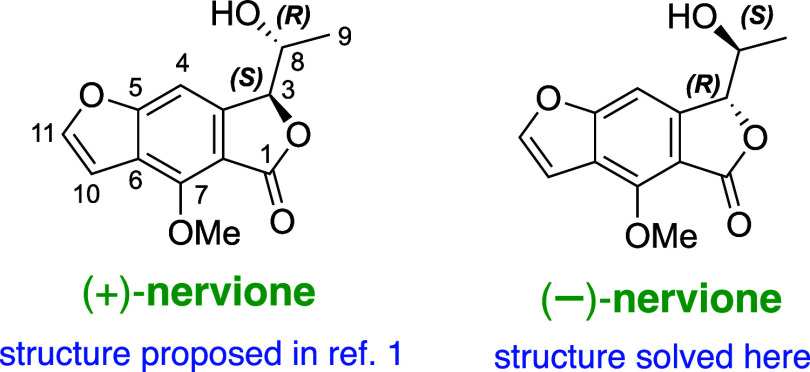
Chemical structure of nervione, as proposed by Duong et al.,^1^ compared with the corrected structural assignment presented in this study.

Nervione, a benzofuran-derivatized natural product newly identified in *Nervilia concolor*, a plant indigenous to Vietnam and prevalent across the tropical and subtropical regions of Asia and the Pacific, was first isolated recently.^1^ Traditionally, *N. concolor* has been utilized in Chinese medicine to treat a variety of ailments including stomatitis, acute pneumonia, bronchitis, and laryngitis.^2^ Despite the extensive use of nervione in traditional medicine, its bioactive properties have not been thoroughly explored. The relative configuration of nervione, illustrated in Figure 1, was determined using computational methods, specifically DFT-NMR chemical shift calculations paired with DP4+ probability analysis.^1,3^ However, its absolute configuration was initially inferred by comparing its electronic circular dichroism (ECD) spectrum with those of previously reported compounds.^4−6^ Specifically, the absolute configuration at the C-3 stereogenic center of nervione was assigned based on its similarity to the positive Cotton effect around 215 nm observed in pestaphthalides A and virgatolides A-B.^5,6^ Like these compounds, nervione displayed a similar positive Cotton effect, leading researchers to assign an *S*-configuration to the C-3 center of nervione. This method, reliant on comparative analysis, still lacks direct experimental validation, underscoring the ongoing challenge of conclusively determining nervione’s structure.^1^ Consequently, nervione merits further investigation through total synthesis to fully understand its structure and biological activities. Herein, we report the first total synthesis and the corrected structural assignment of this natural product, nervione.

## Results and Discussion

**Scheme 1:**
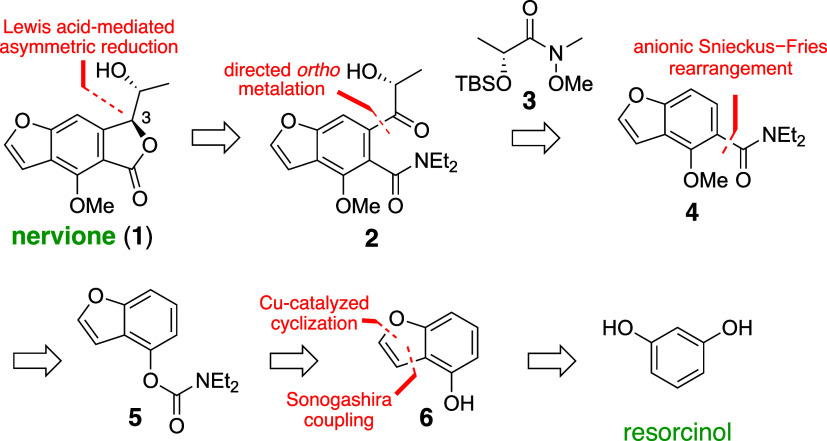
Retrosynthetic Analysis of Nervione

As depicted in Scheme 1, our retrosynthetic strategy involves a late-stage, Lewis acid-mediated diastereoselective reduction of intermediate **2**, which is pivotal for establishing the stereochemistry at the C-3 of nervione (**1**). The stereoinduction is anticipated to be influenced by the α-hydroxy ketone moiety, which is incorporated through the reaction of Weinreb amide **3** with intermediate **4** via directed *ortho*-metalation. Intermediate **4** is accessible through *ortho*-directed strategies coupled with furan formation, a protocol established in our previous work.^7,8^ Consequently, commercially available resorcinol was selected as the starting point for our synthesis pathway.

**Scheme 2:**
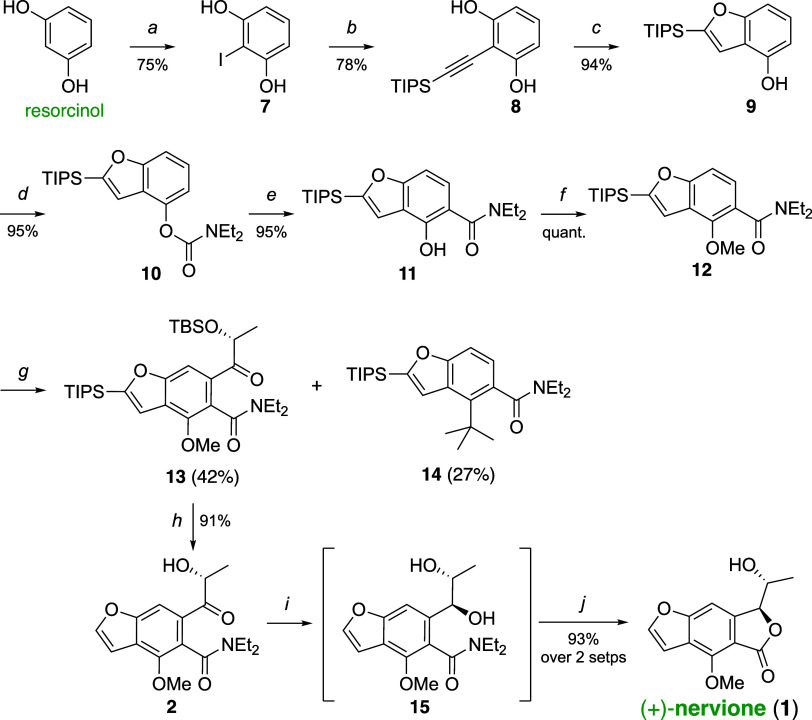
Total Synthesis of (+)-Nervione *Reagents and conditions*: (a) I_2_, NaHCO_3_, H_2_O, 0 °C, rt, 30 min; (b) triisopropylsilylacetylene, Pd(PPh_3_)_2_Cl_2_, CuI, Et_3_N, 1,4-dioxane, 60 °C, 2 h; (c) CuCl, Cs_2_CO_3_, MeCN, 80 °C, 15 h; (*d*) *N,N*-diethylchloroformamide, K_2_CO_3_, MeCN, reflux, 19 h; (e) LiTMP, THF, −78 °C, 1 h, then −20 °C for 4 h; (f) K_2_CO_3_, MeI, acetone, reflux, 20 h; (g) *t*-BuLi, TMEDA, THF, −78 °C, 1 h, then (*R*)-**3**, 2 h; (h) TBAF, THF, 0 °C, 30 min; (i) SnCl_4_, BH_3_·py, CH_2_Cl_2_, −78 °C, 15 min; (j) KOAc, *o*-xylene, 130 °C, 4 h.

Accordingly, resorcinol was initially converted to 2-iodoresorcinol (**7**) through established procedures,^9^ achieving a 75% yield (Scheme 2). Compound **7** then underwent Sonogashira coupling with triisopropylsilylacetylene, yielding coupling adduct **8** in 78% yield. This intermediate was subsequently cyclized into benzofuran **9** through Cu(I)-catalyzed intramolecular hydroalkoxylation,^10^ affording a high 94% yield. The reaction sequence proceeded with the treatment of **9** with *N*,*N*-diethylchloroformamide under basic conditions in refluxing acetonitrile, forming carbamate **10**. Deprotonation of **10** using lithium tetramethylpiperidide (LiTMP) in tetrahydrofuran (THF) at −78 °C led to the formation of the desired Fries product **11**. The choice of LiTMP as the base and the steric hindrance provided by the TIPS group at the C-2 position were critical in suppressing undesired ring opening of the furan, a process typically initiated by deprotonation at the 3-position of the benzofuran.^11^

**Scheme 3:**
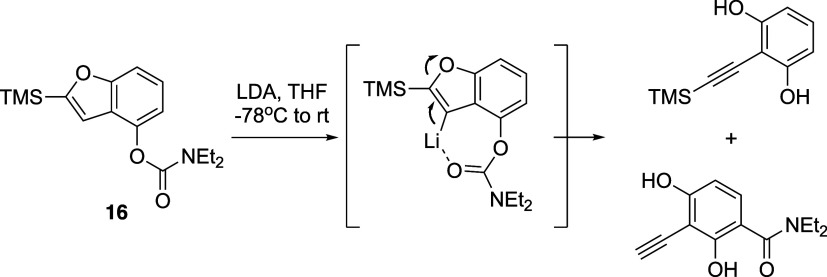
Base-Mediated Opening of the Furan Ring Initiated by Deprotonation at the 3-Position of the Benzofuran, Directed and Facilitated by the Directing Group

As shown in Scheme 3, exposure of the TMS analog **16** to standard rearrangement conditions resulted in a mixture of ring-opening products,^12^ underscoring the importance of the steric effect afforded by the TIPS group and LiTMP base. The proposed mechanism for the base-mediated furan ring opening involves deprotonation at the 3-position of the benzofuran, a step facilitated by the directing group, which stabilizes the resulting carbanion. This intermediate then undergoes ring-opening through cleavage of the C–O bond in the furan moiety. This tactically modified Snieckus–Fries rearrangement significantly enhanced the efficiency of the transformation, achieving an excellent 95% yield for product **11**.

Further synthetic elaboration included methylation of the phenolic hydroxyl group using methyl iodide. Directed *ortho*-metalation of benzofuran **12** was then achieved using *t-*BuLi and *N,N,N′,N′*-tetramethylethylenediamine (TMEDA) in THF at −78 °C, yielding an aryl lithium species. This species was subsequently reacted with Weinreb amide (*R*)-**3** to afford intermediate **13** in moderate yield. The reduced yield can be attributed to a competing nucleophilic aromatic substitution (S_N_Ar) of *t*-BuLi at the benzofuran **12**, leading to the formation of a side product, 4-(*tert*-butyl)-*N,N*-diethyl-2-(triisopropylsilyl)benzofuran-5-carboxamide (**14**) (also see Supporting Information).^13^

**Scheme 4:**
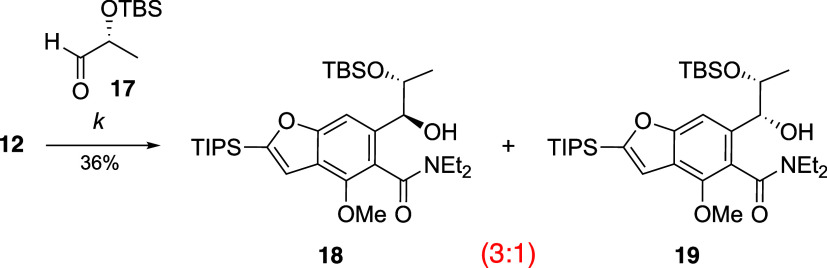
Nucleophilic Addition of Lithiated **12** to (*R*)-Aldehyde **17** *Reagents and conditions*: (*k*) *t*-BuLi, TMEDA, THF, −78 °C, 1 h, then (*R*)-**17**, 2 h.

An attempt was also made to achieve the nucleophilic addition of lithiated **12** to aldehyde (*R*)-**17**; however, the unsatisfactory substrate-controlled diastereoselectivity, yielding a mixture of diastereomers **18** and **19** in a 3:1 ratio, hindered the subsequent steps in the synthesis (Scheme 4).^14^ Given this limitation, we then maintained our focus on the reaction of intermediate **13**.

Upon silyl deprotection with TBAF, the resultant compound aligned with the planned intermediate **2** from our retrosynthetic analysis. For the pivotal Lewis acid-mediated diastereoselective reduction, various chelated and nonchelated reducing conditions were evaluated, as shown in Table 1.^15^ The diol products were then subjected to directed cyclization, yielding nervione and its diastereomer, facilitating a more straightforward characterization. As detailed in Table 1, conventional reduction agents like sodium borohydride, diisobutylaluminum hydride (DIBALH), zinc borohydride, and borane-pyridine complex were employed to reduce intermediate **2**, yielding nervione and its diastereomer **1′** with moderate yields (60–79%) but limited selectivity (entries 1–4). An enhancement in diastereoselectivity to a 7:1 ratio with an 87% yield was observed when titanium tetrachloride was introduced into the borane-pyridine complex reduction of **2** (entry 5).^15d,15f^ A significant improvement was achieved with the use of tin tetrachloride, where the desired (+)-nervione ([α]_D_^23^ = +76.2) product predominated, achieving a diastereoselectivity ratio exceeding 20:1 (entry 6) in 93% yield. Notably, the reaction solvent (dichloromethane) and the borane carrier, pyridine, which may also function as a base facilitating the coordination of the hydroxy group to the tin center, played a crucial role in achieving the high level of diastereoselectivity observed (entries 7–10; see Supporting Information for further details).

**Table 1:**
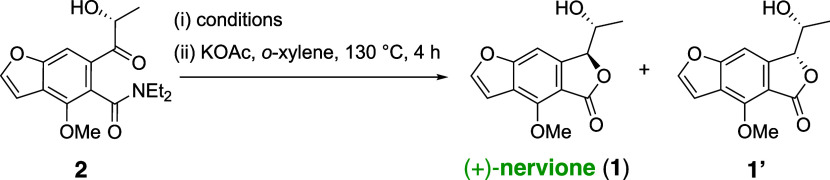
Lewis Acid-Mediated Diastereoselective Reduction of **2**

entry^a^	Lewis acid	reductant	solvent	d*r* (1:1′)^b^	yield^c^
1		NaBH_4_	MeOH	2:1	69^d^
2		DIBALH	toluene	0.5:1	60^e^
3		Zn(BH_4_)_2_	CH_2_Cl_2_	3.4:1	71^f^
4		BH_3_·py^g^	CH_2_Cl_2_	0.9:1	79
5	TiCl_4_	BH_3_·py	CH_2_Cl_2_	7:1	87
6	**SnCl**_**4**_	**BH**_**3**_**·py**	**CH**_2_**Cl**_2_	>20:1	**93**
7	SnCl_4_	BH_3_·py	toluene	2.3:1	83
8	SnCl_4_	BH_3_·py	THF	0.8:1	81
9	SnCl_4_	BH_3_·THF	CH_2_Cl_2_	1.2:1	84
10	SnCl_4_	BH_3_·SMe_2_	CH_2_Cl_2_	0.9:1	65^h^

a *Reagents and conditions*: **2** (0.05 mmol), Lewis acid (1.5 equiv), reducing agent (1.5 equiv), solvent (0.5 mL), −78 °C.

b Determined by ^1^H NMR.

c Isolated yield of **1** and **1′**.

d NaBH_4_ (4.0 equiv), MeOH (0.35 mL), 0 °C, 1 h.

e DIBALH (1.0 equiv), toluene (0.15 mL), −78 °C.

f Zn(BH_4_)_2_ (1.0 equiv), CH_2_Cl_2_ (0.15 mL), 0 °C to rt, 1 h.

g py = pyridine.

h 3 h.

**Figure 2:**
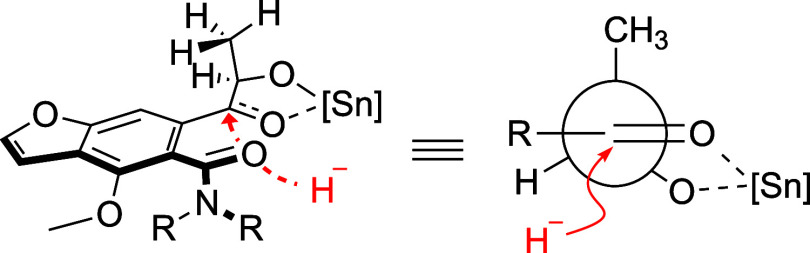
Proposed transition state model for the Lewis acid-mediated diastereoselective reduction of compound **2**, illustrating how the coordination with SnCl_4_ directs the stereoselectivity of the hydride addition.

Figure 2 presents a proposed transition state model for the Lewis acid-mediated diastereoselective reduction of compound **2**.^15b^ In this model, SnCl_4_ coordinates with the two oxygen atoms of the α-hydroxy ketone, creating a well-defined arrangement that facilitates the selective attack of the hydride on the ketone group. The stereoselectivity of the hydride attack is guided by both steric and electronic effects induced by the SnCl_4_ coordination. Specifically, the methyl group effectively blocks the *si* face of the ketone, directing the reducing agent toward the *re* face, resulting in high diastereoselectivity. The simplified version of the model further illustrates the spatial relationships between the functional groups and the SnCl_4_ complex, emphasizing the key factors that influence the stereochemical outcome of the reduction process. In addition, the absence of optical rotation in the authentic nervione isolated prompted us to synthesize (−)-nervione for definitive verification. (−)-Nervione was synthesized through the same synthetic pathway but by utilizing Weinreb amide (*S*)-**3** instead of (*R*)-**3**. This approach successfully produced (−)-nervione with an optical rotation of [α]_D_^23^ = – 77.2.

**Figure 3:**
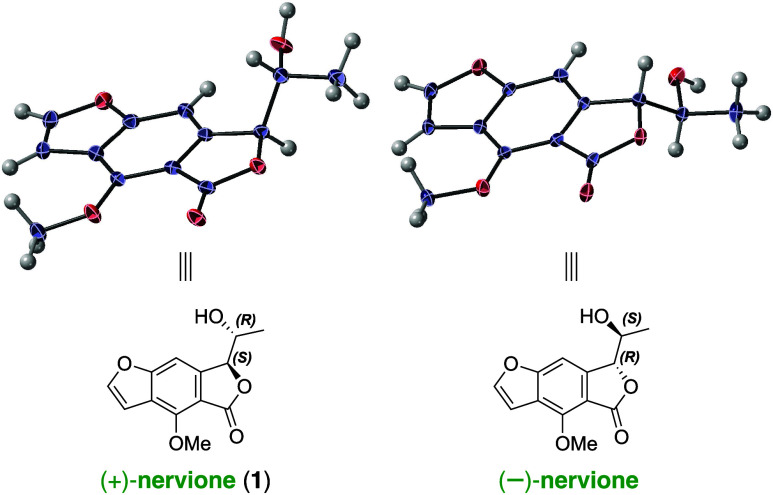
Verification of the absolute configuration of (+)-nervione and (−)-nervione using X-ray crystallography. Displacement ellipsoids are drawn at the 50% probability level (CCDC-2340879 and CCDC-2374994).

**Figure 4:**
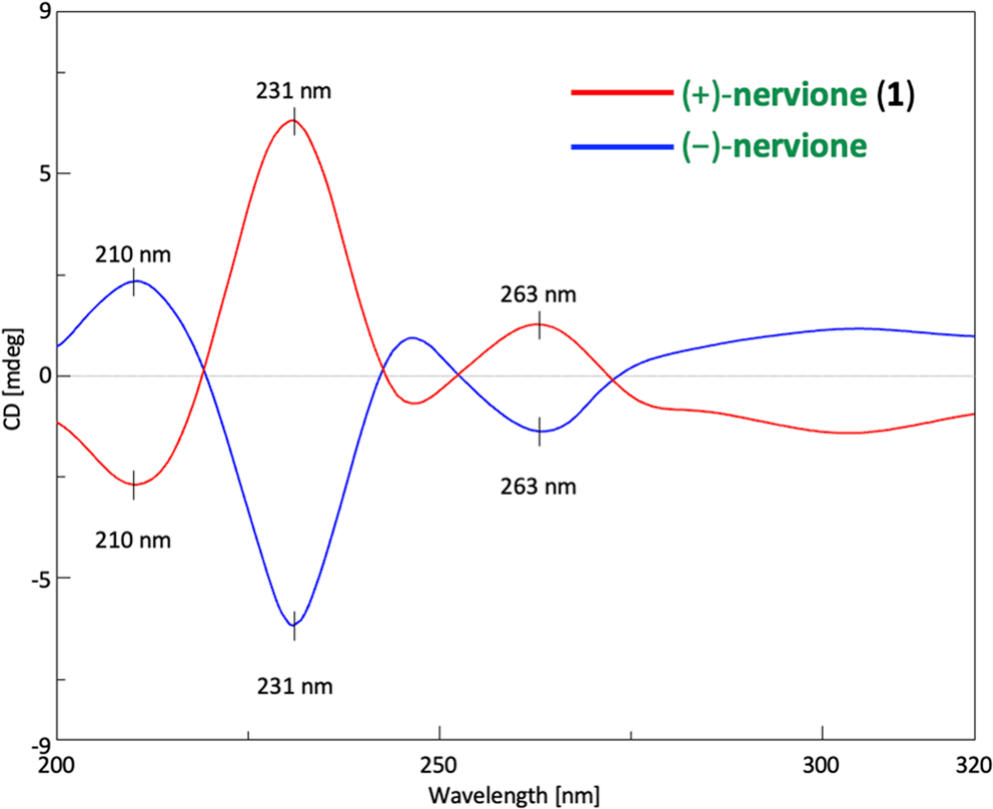
Comparison of the CD spectra for compounds (+)-nervione (**1**) (red) and (−)-nervione (blue), where the CD of nature product of nervione is the same as that of (−)-nervione.

The ^1^H and ^13^C NMR spectra of both synthesized (+)-nervione and (−)-nervione are consistent with those of the natural product isolated from *N. concolor*. X-ray crystallographic analysis unambiguously confirmed the absolute configurations of (+)-nervione and (−)-nervione, as depicted in Figure 3. Figure 4 presents the circular dichroism (CD) spectra for both (+)-nervione and (−)-nervione, demonstrating opposite dichroic absorptions that confirm the optical purity of the products. Detailed analysis reveals that the CD spectrum of (−)-nervione exhibits a positive Cotton effect near 210 nm, in agreement with the properties of the natural product nervione reported by Duong et al.^1^ Contrarily, Duong et al. initially suggested their isolated structure as (+)-nervione. Given that the optical configuration has been unequivocally established through X-ray crystallography in this study, we have reassigned the natural product nervione to the (−)-nervione configuration.

## Conclusions

In this study, we have successfully demonstrated the asymmetric total synthesis of nervione in both its optically active (+) and (−) forms, underscoring the molecular feasibility and stereochemical adaptability of our synthetic methodology. Beginning with commercially available resorcinol, we crafted a robust synthetic route that encompassed ten steps, highlighted by *ortho*-directed strategies and a pivotal late-stage, Lewis acid-mediated, highly diastereoselective reduction. A significant achievement of our research was the resolution of discrepancies in the CD spectra between the synthesized nervione and its naturally isolated counterpart. Initially characterized as (3*S*, 8*R*), the absolute configuration of naturally occurring nervione was revised to (3*R*, 8*S*) based on our synthetic findings. Overall, our results contribute profoundly to the field of natural product synthesis by offering a reliable method for constructing natural molecules and deepening our understanding of nervione’s structural complexities. The techniques developed in this work are expected to promote further investigations into the biological properties of nervione and similar compounds, potentially broadening their therapeutic and industrial applications.

## Experimental Section

### General information

The reactions were performed in the flame-dried glassware under dry nitrogen pressure unless mentioned otherwise, and standard Schlenk techniques were followed. Solvents were freshly prepared by an Innovative technology solvent drying system. The reactions were monitored by TLC using TLC glass plates precoated with silica gel 60 F_254_ (Merck). TLC was visualized with UV, KMnO_4_ stain, or ammonium molybdate stain. Optical rotation values were measured with a Jasco P-2000 polarimeter. IR spectra were recorded with a Thermo Nicolet iS-5FT-IR spectrophotometer. Column chromatography was performed on silica gel Geduran Si 60 (230–400 mesh) (Merck). ^1^H and ^13^C{^1^H} NMR spectra were recorded on a Bruker AV400 MHz, AVIII-400 MHz, and AV-500 MHz spectrometers in CDCl_3_ or CD_3_OD. NMR chemical shifts were reported in ppm and were measured relative to CHCl_3_ (7.26 ppm for ^1^H and 77.16 ppm for ^13^C{^1^H}) or CH_3_OH (3.31 ppm for ^1^H and 49.00 ppm for ^13^C{^1^H}). Abbreviations in the NMR data are s = singlet, d = doublet, t = triplet, br = broad, dd = doublet of doublet, qd = quartet of doublet, q = quartet, quint = quintet, hept = heptet, m = multiplet. HR-ESI mass spectra were conducted on a JMS-T100LP AccuTOF LC-plus 4G TOF mass spectrometer (JEOL, Tokyo, Japan). HR-EI and HR-FAB mass spectra were conducted on a JMS-700 double-focusing magnetic sector mass spectrometer (JEOL, Tokyo, Japan) with a resolution of 8000(3000) (5% valley definition). For FAB mass spectra, the source accelerating voltage was operated 10 kV with Xe gun, using 3-nitrobenzyl alcohol (NBA) as a matrix. Circular dichroism spectra were recorded on JASCO J-815 CD Spectropolarimeter. Melting points were recorded on the Buchi M-565 apparatus. Single-crystal X-ray diffraction was measured in Bruker D8 Venture SC-XRD. 2-iodoresorcinol **7**,^9^ Weinreb amide,^16^ and (*R*)-**17**^17^ were prepared according to the literature.

#### Procedures for the Synthesis of 2-Iodobenzene-1,3-diol (**7**)^9^

To a solution of resorcinol (550.1 mg, 5.0 mmol) and I_2_ (1.39 g, 5.5 mmol) in water (5.0 mL) was cooled at 0 °C in an ice bath, NaHCO_3_ was slowly added, and vigorously stirred at 0 °C for 30 min. After warming up to room temperature, the reaction was stirred for 10 min. The precipitate was filtrated off, and the filtrate was extracted with EtOAc (3 × 30 mL). The combined organic layer was dried over anhydrous Na_2_SO_4_, filtered, and concentrated under reduced pressure. The crude product was purified by column chromatography (silica gel, 10% EtOAc/Hexane) to afford 2-Iodobenzene −1,3-diol (**7**) (891.2 mg, 75%) as a white solid. ^1^H NMR (400 MHz, CDCl_3_) δ: 7.11 (t, *J* = 8.1 Hz, 1H), 6.56 (d, *J* = 8.1 Hz, 2H), 5.29 (br s, 2H); ^13^C{^1^H} NMR (100 MHz, CDCl_3_) δ: 155.8, 130.5, 107.5, 77.8.

#### Procedures for the Synthesis of 2-((Triisopropylsilyl)ethynyl)benzene-1,3-diol (**8**)

To a solution of 2-iodobenzene-1,3-diol **7** (944.0 mg, 4.0 mmol) in dry 1,4-dioxane (10.0 mL) under nitrogen atmosphere, Pd(PPh_3_)_2_Cl_2_ (140.4 mg, 0.2 mmol), and CuI (76.2 mg, 0.4 mmol) were added. Then Et_3_N (1.68 mL, 12 mmol), and triisopropylsilyl acetylene (1.8 mL, 8.0 mmol) were added to the mixture, respectively. The mixture was stirred at 60 °C in an oil bath for 2 h. After completion, the reaction mixture was filtrated through Celite and concentrated under reduced pressure. The crude product was purified by column chromatography (silica gel, 5% EtOAc/Hexane) to afford the 2-((triisopropylsilyl)ethynyl)benzene-1,3-diol (**8)** (906.7 mg, 78%) as a pale yellow solid; mp = 38.9 - 39.9 °C; FTIR (neat): 3497, 2943, 2865, 2140, 1464, 1185, 1013, 783, 724, 677 cm^–1^; ^1^H NMR (400 MHz, CDCl_3_) δ: 7.13 (t, *J* = 8.4 Hz, 1H), 6.52 (d, *J* = 8.4 Hz, 2H), 5.53 (br s, 2H), 1.21–1.12 (m, 21H); ^13^C{^1^H} NMR (100 MHz, CDCl_3_) δ: 157.4, 131.1, 106.8, 106.3, 98.7, 95.7, 18.8, 11.2; HRMS (FAB+) *m*/*z*: [M + H]^+^ Calcd for C_17_H_27_O_2_Si 291.1775; Found 291.1773.

#### Procedures for the Synthesis of 2-(Triisopropylsilyl)benzofuran-4-ol (**9**)

A 2-((triisopropylsilyl)ethynyl)benzene-1,3-diol **8** (725.0 mg, 2.5 mmol), CuCl (12.3 mg, 0.125 mmol), and Cs_2_CO_3_ (40.7 mg, 0.125 mmol) were dissolved in dry MeCN (8.4 mL) under nitrogen atmosphere. The mixture was freeze–pump–thaw 3 cycles to remove residual gases. Then, the mixture was stirred at 80 °C in a heating block for 15 h. After completion, the reaction mixture was concentrated under reduced pressure. The crude product was purified by column chromatography (silica gel, 5% EtOAc/Hexane) to afford 2-(triisopropylsilyl)benzofuran-4-ol (**9)** (682.5 mg, 94%) as a white solid; mp = 93.3 - 94.5 °C; FTIR (neat): 3239, 2959, 2939, 2868, 1249, 1077, 1014, 882, 812, 709, 635 cm^–1^; ^1^H NMR (400 MHz, CDCl_3_) δ: 7.15–7.09 (m, 3H), 6.60 (dd, *J* = 6.0, 2.4 Hz, 1H), 1.39 (hept, *J* = 7.4 Hz, 3H), 1.15 (d, *J* = 7.4 Hz, 18H); ^13^C{^1^H} NMR (100 MHz, CDCl_3_) δ: 160.0, 159.6, 149.0, 124.9, 117.4, 114.5, 107.3, 104.7, 18.7, 11.2; HRMS (FAB+) *m*/*z*: [M]^+^ Calcd for C_17_H_26_O_2_Si 290.1702; Found 290.1705.

#### Procedures for the Synthesis of 2-(Triisopropylsilyl)benzofuran-4-yl Diethylcarbamate (**10**)

A suspension of 2-(triisopropylsilyl)benzofuran-4-ol **9** (580.3 mg, 2.0 mmol) and K_2_CO_3_ (414.6 mg, 3.0 mmol) in dry MeCN (0.75 mL) was added diethylcarbamyl chloride (0.42 mL, 3.0 mmol), and the mixture was refluxed for 19 h in a heating block under a nitrogen atmosphere. Upon completion, the mixture was quenched with H_2_O and extracted with EtOAc (3 × 20 mL). The combined organic layers were dried over anhydrous Na_2_SO_4_, filtered, and concentrated under reduced pressure. The crude product was purified by column chromatography (silica gel, 5% EtOAc/Hexane) to afford 2-(triisopropylsilyl)benzofuran-4-yl diethylcarbamate (**10)** (739.5 mg, 95%) as a white solid. mp = 40.1 - 41.1 °C; FTIR (neat): 2960, 2943, 2891, 2866, 1727, 1416, 1258, 1234, 1154, 1076 cm^–1^; ^1^H NMR (400 MHz, CDCl_3_) δ: 7.35 (d, *J* = 8.4 Hz, 1H), 7.23 (t, *J* = 8.0 Hz, 1H), 7.01 (d, *J* = 8.0 Hz, 1H), 6.92 (s, 1H), 3.53 – 3.43 (m, 4H), 1.43 – 1.25 (m, 9H), 1.14 (d, *J* = 7.4 Hz, 18H); ^13^C{^1^H} NMR (100 MHz, CDCl_3_) δ: 160.6, 159.6, 153.9, 144.4, 124.4, 121.9, 115.0, 114.8, 108.4, 42.5, 42.3, 18.7, 14.5, 13.6, 11.2; HRMS (ESI) *m*/*z*: [M + H]^+^ Calcd for C_22_H_36_NO_3_Si 390.2459; Found 390.2461.

#### Procedures for the Synthesis of *N,N*-Diethyl-4-hydroxy-2-(triisopropylsilyl)benzofuran-5-carboxamide (**11**)

##### Preparation of LiTMP

A solution of 2,2,6,6-tetramethylpiperidine (1.08 mL, 6.24 mmol, dried over 4 Å molecular sieve and distilled) in dry THF (3.0 mL) was cooled at 0 °C. Then, *n*-BuLi (1.97 M in hexane, 3.05 mL, 6.0 mmol) was added dropwise. The mixture was stirred at 0 °C in an ice bath for 10 min. To a Schlenk tube, a solution of 2-(triisopropylsilyl)benzofuran-4-yl diethylcarbamate **10** (583.9 mg, 1.5 mmol) in dry THF (5.0 mL) was cooled at −78 °C. Then, LiTMP (0.84 M to THF, 6.25 mL, 5.25 mmol) was added to the solution dropwise and stirred at −78 °C for 1 h. After that, the mixture was gradually warmed up to −20 °C and stirred for 4 h. Upon completion, saturated NH_4_Cl (15.0 mL) was added. The mixture was extracted with EtOAc (3 × 20 mL). The combined organic layers were dried over anhydrous Na_2_SO_4_, filtrated, and concentrated *in vacuo*. The crude product was purified by column chromatography (silica gel, 5% EtOAc/Hexane) to afford *N,N*-diethyl-4-hydroxy-2-(triisopropylsilyl)benzofuran-5-carboxamide (**11)** (554.8 mg, 95%) as a white solid. mp = 148.2–149.6 °C; FTIR (neat): 3125, 2943, 2866, 1589, 1462, 1316, 1279, 1082, 883, 679, 655 cm^–1^; ^1^H NMR (400 MHz, CDCl_3_) δ: 7.22 – 7.21 (m, 2H), 6.99 (d, *J* = 8.6 Hz, 1H), 3.56 (q, *J* = 7.1 Hz, 4H), 1.42 – 1.35 (m, 3H), 1.30 (t, *J* = 7.1 Hz, 6H), 1.14 (d, *J* = 7.4 Hz, 18H); ^13^C{^1^H} NMR (100 MHz, CDCl_3_) δ: 172.8, 160.8, 160.3, 154.8, 123.8, 118.7, 116.1, 110.3, 102.4, 42.4, 18.7, 13.6, 11.2; HRMS (ESI) *m*/*z*: [M + H]^+^ Calcd for C_22_H_36_NO_3_Si 390.2459; Found 390.2460.

#### Procedures for the Synthesis of *N,N*-Diethyl-4-methoxy-2-(triisopropylsilyl)benzofuran-5-carboxamide (**12**)

To a suspension of *N,N*-diethyl-4-hydroxy-2-(triisopropylsilyl)benzofuran-5-carboxamide **11** (389.3 mg, 1.0 mmol), and K_2_CO_3_ (165.9 mg, 1.2 mmol) in acetone (10.0 mL) was added MeI (74.8 μL, 1.2 mmol) and reflux for 20 h in a heating block under air atmosphere. After completion, acetone was evaporated, H_2_O (15.0 mL) was added, and the mixture was extracted with EtOAc (3 × 15 mL). The combined organic layers were dried over anhydrous Na_2_SO_4_, filtrated, and concentrated *in vacuo*. The crude product was purified by column chromatography (silica gel, 30% EtOAc/Hexane) to afford *N,N*-diethyl-4-methoxy-2-(triisopropylsilyl)benzofuran-5-carboxamide (**12)** (400.7 mg, 99%) as a white solid. mp = 39.9 - 42.0 °C; FTIR (neat): 2965, 2943, 2887, 2866, 1624, 1467, 1422, 1298, 1085, 883, 681 cm^–1^; ^1^H NMR (400 MHz, CDCl_3_) δ: 7.21 (d, *J* = 8.3 Hz, 1H), 7.12 (s, 1H), 7.08 (d, *J* = 8.3 Hz, 1H), 4.07 (s, 3H), 3.59 (br d, *J* = 75.2 Hz, 2H), 3.30–3.09 (m, 2H), 1.40 (hept, *J* = 7.4 Hz, 3H), 1.26 (t, *J* = 7.1 Hz, 3H), 1.14 (d, *J* = 7.5 Hz, 17H), 1.04 (t, *J* = 7.1 Hz, 3H); ^13^C{^1^H} NMR (100 MHz, CDCl_3_) δ: 169.5, 160.6, 160.0, 149.1, 123.6, 122.3, 119.5, 115.8, 106.5, 60.7, 43.2, 39.1, 18.7, 14.02 12.9, 11.1. HRMS (ESI+) *m*/*z*: [M + H]^+^ Calcd for C_23_H_38_NO_3_Si 404.2615; Found 404.2611.

#### (*R*)-2-((*tert*-Butyldimethylsilyl)oxy)-*N*-methoxy-*N*-methylpropanamide ((*R*)-**3**)^16^

^1^H NMR (400 MHz, CDCl_3_) δ: 4.68 – 4.63 (m, 1H), 3.68 (s, 3H), 3.19 (s, 3H), 1.33 (d, *J* = 6.6 Hz, 3H), 0.88 (s, 9H), 0.08 (s, 3H), 0.05 (s, 3H); ^13^C{^1^H} NMR (100 MHz, CDCl_3_) δ: 175.1, 66.7, 61.3, 32.9, 25.9, 21.0, 18.4, −4.5, −4.8.

#### Procedures for the Synthesis of (*R*)-6-(2-((*tert*-Butyldimethylsilyl)oxy)propanoyl)-*N,N*-diethyl-4-methoxy-2-(triisopropylsilyl)benzofuran-5-carboxamide (**13**)

To a solution of *N,N*-diethyl-4-methoxy-2-(triisopropylsilyl)benzofuran-5-carboxamide **12** (203.1 mg, 0.5 mmol) and TMEDA (118.5 μL, 0.79 mmol) in THF (1.7 mL) at −78 °C under nitrogen atmosphere was added *t*-BuLi (1.56 M in pantane, 0.48 mL, 0.75 mmol) dropwise. The mixture was stirred for 1 h. Then the (*R*)-2-((*tert*-butyldimethylsilyl)oxy)-*N*-methoxy-*N*-methylpropanamide ((*R*)-**3**) (197.6 μL, 0.75 mmol) was added to the reaction mixture dropwise and stirred at −78 °C for 2 h. Upon completion, the reaction was quenched by saturated NH_4_Cl at −78 °C and extracted with EtOAc (3 × 15 mL). The combined organic layers were dried over anhydrous Na_2_SO_4_, filtrated, and concentrated *in vacuo*. The crude product was purified by column chromatography (silica gel, 10% EtOAc/DCM) to afford (*R*)-6-(2-((*tert*-butyldimethylsilyl)oxy)propanoyl)-*N,N-*diethyl-4-methoxy-2-(triisopropylsilyl)benzofuran-5-carboxamide (**13)** (124.5 mg, 42%) as a white solid. mp = 90.0 - 91.5 °C; FTIR (neat): 2945, 2891, 2866, 1701, 1126, 1461, 1316, 1291, 1100, 833 cm^–1^; ^1^H NMR (400 MHz, CDCl_3_, rotamer) δ: 8.14 and 7.82 (2 s, 1H, rotamer), 7.14 (s, 1H), 4.90 and 4.80 (2 q, *J* = 6.8 Hz, 1H, rotamer), 4.07 and 4.06 (2 s, 3H, rotamer), 3.67 – 3.52 (m, 2H), 3.18 and 3.11 (2 q, *J* = 7.2 Hz 2H, rotamer), 1.51 and 1.49 (2 d, *J* = 6.9 Hz, 3H, rotamer), 1.45 – 1.36 (m, 3H), 1.32 – 1.27 (m, 3H), 1.15 (dd, *J* = 7.5, 2.0 Hz, 18H), 1.08 and 1.03 (2 t, *J* = 7.5, 2.0 Hz, 3H, rotamer), 0.88 and 0.84 (2 s, 9H), 0.10 and 0.08 (2 s, 3H, rotamer), 0.06 and 0.01 (2 s, 3H, rotamer); ^13^C{^1^H} NMR (100 MHz, CDCl_3_, rotamer) δ: 202.0 and (200.6), (168.1) and 167.8, (164.6) and 164.2, (158.5) and 158.4, (149.5) and 149.4, 131.0 and (129.4), (124.0) and 123.8, (122.9) and 122.3, 115.9 and (115.8), (109.1) and 108.2, (75.4) and 73.7, (61.1) and 61.0, 43.1 and (43.0), (38.9) and 38.7, 25.9 and (25.8), (22.2) and 21.9, 18.6 and (18.3), (13.6) and 13.4, (12.4) and 11.1, −4.6 and (−4.7); HRMS (ESI+) *m*/*z*: [M + H]^+^ Calcd for C_32_H_56_NO_5_Si_2_ 590.3692; Found 590.3685.

#### 4-(*tert*-Butyl)-*N,N*-diethyl-2-(triisopropylsilyl)benzofuran-5-carboxamide (**14**)

(57.9 mg, 27%) as colorless liquid; ^1^H NMR (500 MHz, CDCl_3_) δ: 7.36 (d, *J* = 8.4 Hz, 1H), 7.24 (s, 1H), 6.92 (d, *J* = 8.4 Hz, 1H), 3.85–3.78 (m, 1H), 3.36–3.25 (m, 2H), 3.13–3.06 (m, 1H), 1.53 (s, 9H), 1.40 (hept, *J* = 7.5 Hz, 3H), 1.24 (t, *J* = 7.1 Hz, 3H), 1.14 (d, *J* = 7.4 Hz, 18H), 1.07 (t, *J* = 7.1 Hz, 3H); ^13^C{^1^H} NMR (125 MHz, CDCl_3_) δ: 173.8, 159.1, 158.3, 139.3, 129.5, 126.9, 124.4, 120.5, 109.9, 43.4, 38.6, 37.9, 31.4, 18.7, 13.3, 11.9, 11.2; HRMS (ESI+) *m*/*z*: [M + H]^+^ Calcd for C_26_H_44_NO_2_Si 430.3136; Found 430.3134.

**Note:** To the synthesis of (*S*)-6-(2-((*tert*-butyldimethylsilyl)oxy)propanoyl)-*N,N*-diethyl-4-methoxy-2-(triisopropylsilyl)benzofuran-5-carboxamide, the (*S*)-2-((*tert*-butyldimethylsilyl)oxy)-*N*-methoxy-*N*-methylpropanamide was use as the reagent.

The reaction between compound **12** and aldehyde (*R*)-**17** was conducted using the same procedure, with the sole modification being the substitution of compound (*R*)-**3** with (*R*)-**17**

#### Procedures for the Synthesis of (*R*)-*N,N*-Diethyl-6-(2-hydroxypropanoyl)-4-methoxybenzofuran-5-carboxamide (**2**)

To a solution of (*R*)-6-(2-((*tert*-butyldimethylsilyl)oxy)propanoyl)-*N,N*-diethyl-4-methoxy-2-(triisopropylsilyl)benzofuran-5-carboxamide **13** (88.3 mg, 0.15 mmol) in THF (1.0 mL) at 0 °C in an ice bath under nitrogen atmosphere was added TBAF (1.0 M in THF, 0.23 mL, 0.225 mmol) dropwise. Then, the reaction mixture was stirred at the same temperature for 30 min. After the reaction was completed, the reaction mixture was quenched with saturated NH_4_Cl and extracted with EtOAc (3 × 10 mL). The combined organic layers were dried over anhydrous Na_2_SO_4_, filtered, and concentrated under reduced pressure. The crude product was quickly purified by column chromatography (silica gel, 70% EtOAc/Hexane) to afford (*R*)-*N,N*-diethyl-6-(2-hydroxypropanoyl)-4-methoxybenzofuran-5-carboxamide (**2)** (43.52 mg, 91%) as colorless sticky oil. FTIR (neat): 3392, 3153, 3117, 2981, 2936, 2872, 1690, 1613, 1349, 1292, 1066 cm^–1^; ^1^H NMR (400 MHz, CDCl_3_, rotamer) δ: 7.76–7.75 (m, 1H), 7.61 and 7.53 (2 d, *J* = 0.6 Hz, 1H, rotamer), 7.00–6.99 (m, 1H), 5.05–4.98 (m, 1H), 4.09 and 4.08 (2 s, 3H, rotamer), 3.69–3.53 (m, 2H), 3.26–3.02 (m, 2H), 1.46–1.42 (m, 3H), 1.31–1.27 (m, 3H), 1.08 and 0.98 (2 t, *J* = 7.2 Hz, 3H, rotamer); ^13^C{^1^H} NMR (100 MHz, CDCl_3_, rotamer) δ: 202.3 and (202.9), (167.6) and 167.4, (155.8) and 155.7, (150.4) and 150.0, (147.6) and 147.5, 130.4 and (129.4), 123.8 and (123.2), (122.6) and 122.1, (107.4) and 107.0, 105.5 and (105.4), 70.52, (61.0) and 60.7, 43.2 and (42.0), (39.0) and 38.9, (21.9) and 21.5, (13.6) and 13.4, 12.4; HRMS (ESI) *m*/*z*: [M + Na]^+^ Calcd for C_17_H_21_NO_5_Na 342.1312; Found 342.1304.

#### Procedures for the Synthesis of (*S*)-7-((*R*)-1-Hydroxyethyl)-4-methoxybenzo[1,2-b:4,5-c′]difuran-5(*7H*)-one (+)-nervione **1**

The (*R*)-*N,N-*diethyl-6-(2-hydroxypropanoyl)-4-methoxybenzofuran-5-carboxamide **2** (16.0 mg, 0.05 mmol) was dissolved in dry DCM (0.5 mL) under a nitrogen atmosphere and cooled at −78 °C. Then, SnCl_4_ (75.0 μL, 1.0 M in heptane, 0.075 mmol) was added and stirred at the same temperature for 15 min. After that, the complex BH_3_.pyridine (0.075 mmol in 0.05 mL of dry DCM) was added to the reaction mixture dropwise (7.0 μL/15 s) and stirred at −78 °C for another 15 min. Upon the competition, the reaction was quenched with saturated NH_4_Cl and extracted with EtOAc (3 × 10 mL). The combined organic layers were dried over anhydrous Na_2_SO_4_, filtered, and evaporated under reduced pressure. The crude product and KOAc (49.0 mg, 0.5 mmol) in a dram vial were dissolved in *o*-xylene (0.5 mL) under an air atmosphere. The reaction mixture was stirred at 130 °C in a heating block for 4 h. After the reaction was completed, the mixture was quenched with H_2_O and extracted with EtOAc (3 × 5 mL). The combined organic layers were dried over anhydrous Na_2_SO_4_, filtered, and concentrated *in vacuo*. The crude product was purified by column chromatography (silica gel, 50% EtOAc/Hexane) to provide (*S*)-7-((*R*)-1-hydroxyethyl)-4-methoxybenzo[1,2-b:4,5-c′]difuran-5(*7H*)-one **(+)**-**nervione** (11.53 mg, 93%, d.r. >20:1) as a white solid. The white solid was dissolved in EtOAc, filtered through a syringe filter, transferred to a half-sealed 4 mL vial, and kept for 1–2 days to afford white needle crystals. mp = 168.6 - 169.5 °C; FTIR (neat): 3499, 2923, 1742, 1614, 1592, 1486, 1348, 1305, 1088, 757 cm^–1^; ^1^H NMR (500 MHz, CD_3_OD) δ: 7.83 (d, *J* = 2.4 Hz, 1H), 7.39 (s, 1H), 7.27 (dd, *J* = 2.3, 0.8 Hz, 1H), 5.36 (dd, *J* = 4.8, 0.8 Hz, 1H), 4.32 (s, 3H), 4.07–4.02 (m, 1H), 1.20 (d, *J* = 6.4 Hz, 3H); ^13^C{^1^H} NMR (125 MHz, CD_3_OD) δ: 170.9, 162.6, 154.8, 147.3, 147.1, 119.4, 110.1, 107.0, 101.2, 84.6, 70.0, 61.1, 18.1; HRMS (ESI) *m*/*z*: [M + Na]^+^ Calcd for C_13_H_12_O_5_Na 271.0577; Found 271.0570. **[α]**_**D**_^**23**^ = +76.2 (c 0.5, CHCl_3_). For **(−)**-**nervione [α]**_**D**_^**23**^ = −77.2 (c 0.5, CHCl_3_).

#### Procedures for the Synthesis of 2-(Trimethylsilyl)benzofuran-4-yl Diethylcarbamate (**16**)

followed the synthesis route of compound **10**, using 5% EtOAc/Hexane as an eluent to afford 2-(trimethylsilyl)benzofuran-4-yl diethylcarbamate **16** (109.8 mg, 96%) as a colorless liquid. ^1^H NMR (400 MHz, CDCl_3_) δ: 7.36–7.34 (m, 1H), 7.23 (t, *J* = 8.0 Hz, 1H), 6.97 (d, *J* = 8.0 Hz, 1H),. 6.85 (d, *J* = 0.8 Hz, 1H), 3.54–3.41 (m, 4H), 1.34–1.22 (m, 6H), 0.34 (s, 9H); ^13^C{^1^H} NMR (100 MHz, CDCl_3_); δ: 163.6, 159.6, 154.0, 144.5, 124.6, 122.2, 114.9, 113.1, 108.6, 42.5, 42.2, 14.5, 13.6, −1.7, HRMS (ESI) *m*/*z*: [M + H]^+^ Calcd for C_16_H_23_NO_3_Si 306.1520; Found 306.1515.

#### (*R*)-2-((*tert*-Butyldimethylsilyl)oxy)propanal ((*R*)-**17**)^17^

^1^H NMR (500 MHz, CDCl_3_) δ: 9.61 (d, *J* = 1.3 Hz, 1H), 4.09 (qd, *J* = 6.9, 1.2 Hz, 1H), 1.27 (d, *J* = 6.9 Hz, 3H), 0.92 (s, 9H), 0.10 (s, 3H), 0.09 (s, 3H); ^13^C{^1^H} NMR (125 MHz, CDCl_3_) δ: 204.3, 74.0, 31.7, 25.9, 22.8, 18.6, 18.3, 14.2, −4.6, −4.7

## Data Availability

The data underlying this study are available in the published article and its online Supporting Information.

## Supporting Information Available

General Information, experimental details, ^1^H and ^13^C NMR spectra, CD spectrum, and X-ray data for (+)-nervione and (−)-nervione (PDF)
