# Supplementary material for: Asymmetric Total Synthesis and Structural Reassignment of Nervione
Source: J Org Chem. 2025 Mar 19;90(12):4389–96. doi: 10.1021/acs.joc.5c00258 (PMC11959527; doi:10.1021/acs.joc.5c00258)
Supplement: Supplementary file 1 — jo5c00258_si_001.pdf [file jo5c00258_si_001.pdf]

Supporting Information

Asymmetric Total Synthesis and Structural Reassignment of Nervione

Kunita Phakdeeyothin,[a,†] Jian-Liang Li,[b,†] Yi-Tian Hong,[c] and Rong-Jie Chein[a,*]

[a] Institute of Chemistry, Academia Sinica, Taipei, 115201, Taiwan

[b] Department of Chemistry, National Taiwan University, Taipei, 10617, Taiwan

[c] Department of Chemistry, National Taiwan Normal University, Taipei, 11677, Taiwan

[†] These authors have equal contributions.

Rong-Jie Chein E-mail: rjchein@chem.sinica.edu.tw

Table of Contents

1.	General Information	S-3
2.	Supplementary Information	S-4
3.	^{1}H and ^{13}C{^{1}H} Spectra of Products	S-7
4.	Circular Dichroism Spectra	S-23
5.	X-Ray Crystal Structure Analysis	S-25
6.	References	S-41

1. General information

The reactions were performed in the flame-dried glassware under dry nitrogen pressure unless mentioned otherwise, and standard Schlenk techniques were followed. Solvents were freshly prepared by an Innovative technology solvent drying system. The reactions were monitored by TLC using TLC glass plates precoated with silica gel 60 F_{254} (Merck). The visualization of TLC was done with UV, $KMnO_4$ stain, or ammonium molybdate stain. Optical rotation values were measured with a Jasco P-2000 polarimeter. IR spectra were recorded with a Thermo Nicolet iS-5FT-IR spectrophotometer. Column chromatography was performed on silica gel Geduran® Si 60 (230-400 mesh) (Merck). ^{1}H and $^{13}C\{^{1}H\}$ NMR spectra were recorded on a Bruker AV400 MHz, AVIII-400 MHz, and AV-500 MHz spectrometers in $CDCl_3$ or CD_3OD. NMR chemical shifts were reported in ppm and were measured relative to $CHCl_3$ (7.26 ppm for ^{1}H and 77.16 ppm for $^{13}C\{^{1}H\}$) or CH_3OH (3.31 ppm for ^{1}H and 49.00 ppm for $^{13}C\{^{1}H\}$). Abbreviations in the NMR data are s = singlet, d = doublet, t =triplet, br = broad, dd = doublet of doublet, qd = quartet of doublet, q = quartet, quint = quintet, hept = heptet, m = multiplet. HR-ESI mass spectra were conducted on a JMS-T100LP AccuTOF LC-plus 4G TOF mass spectrometer (JEOL, Tokyo, Japan). HR-EI and HR-FAB mass spectra were conducted on a JMS-700 double focusing magnetic sector mass spectrometer (JEOL, Tokyo, Japan) with a resolution of 8000(3000) (5% valley definition). For FAB mass spectra, the source accelerating voltage was operated 10kV with Xe gun, using 3-nitrobenzyl alcohol (NBA) as a matrix. Circular dichroism spectra were recorded on JASCO J-815 CD Spectropolarimeter. Melting points were recorded on the Buchi M-565 apparatus. Single-crystal X-ray diffraction was measured in Bruker D8 Venture SC-XRD. 2-iodoresorcinol **7**[1], Weinreb amide[2], and (*R*)-**17**[3] were prepared according to literature.

2. Supplementary Information

Table S1. Optimization of Sonogashira cross coupling reaction[a]

Entry[a]	TIPS acetylene (equiv)	NEt_3 (equiv)	%Yield[b]
1	1.0	5.0	37
2	1.5	5.0	60
3	2.0	5.0	78
4	3.0	5.0	80
5	2.0	3.0	78

[a]conditions: **7** (0.5 mmol), triisopropylsilyl acetylene, CuI (10 mol%), 1,4-dioxane (1.25 mL), 60 °C, 2 h, [b]Isolated yield.

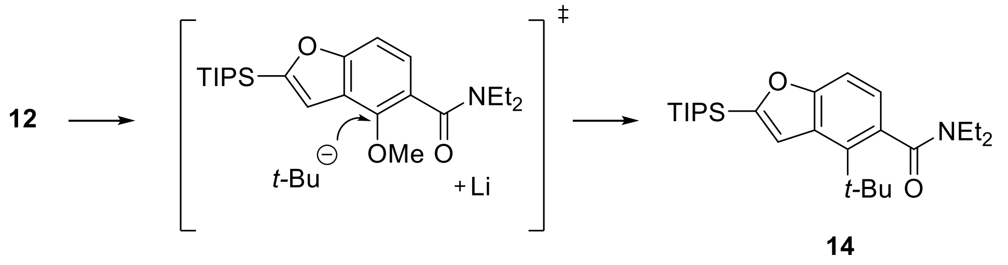

Scheme S1. The formation of compound **14** through the 1,4-addition of *t*-BuLi to compound **12.**[4]

Table S2. Optimization reaction of Lewis acid-mediated diastereoselective reduction of **2**[a]

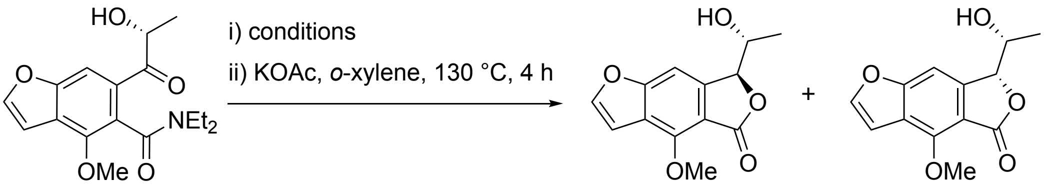

2 (+)-**nervione** (**1**) **1'**

**Entry**[a]	**Lewis acid**	**Reductant**	**Solvent**	**dr (1 : 1')**[b]	**Yield (%)**[c]
1	-	$NaBH_4$	MeOH	2.0 : 1.0	69[d]
2	-	DIBALH (1.0 M in toluene)	toluene	0.5 : 1.0	60[e]
3	-	$Zn(BH_4)_2$ (0.16 M in THF)	CH_2Cl_2	3.4 : 1.0	71[f]
4	-	$BH_3{\cdot}py$[g]	CH_2Cl_2	0.9 : 1.0	79
5	BCl_3 (1.0 M in toluene)	$BH_3{\cdot}py$	CH_2Cl_2	1.7 : 1.0	84
6	$AlCl_3$ (1.0 M in nitrobenzene)	$BH_3{\cdot}py$	CH_2Cl_2	2.9 : 1.0	98
7	$AlMe_3$ (2.0 M in Toluene)	$BH_3{\cdot}py$	CH_2Cl_2	0.9 : 1.0	97
8	$AlMe(BHT)_2$ (0.83 M in CH_2Cl_2)	$BH_3{\cdot}py$	CH_2Cl_2	1.3 : 1.0	82
9	$TiCl_4$ (1.0 M in toluene)	$BH_3{\cdot}py$	CH_2Cl_2	7.0 : 1.0	87
10	$ZrCl_4$	$BH_3{\cdot}py$	CH_2Cl_2	0.9 : 1.0	95
11	$SnCl_4$ (1.0 M in heptane)	$BH_3{\cdot}py$	CH_2Cl_2	>20 : 1.0	93
12	$SnCl_4$ (1.0 M in heptane)	$BH_3{\cdot}py$	toluene	2.3 : 1.0	83
13	$SnCl_4$ (1.0 M in heptane)	$BH_3{\cdot}py$	THF	0.8 : 1.0	81
14	$SnCl_4$ (1.0 M in heptane)	$BH_3{\cdot}THF$	CH_2Cl_2	1.2 : 1.0	84
15	$SnCl_4$ (1.0 M in heptane)	$BH_3{\cdot}SMe_2$	CH_2Cl_2	0.9 : 1.0	43
16	$SnCl_4$ (1.0 M in heptane)	$BH_3{\cdot}SMe_2$	CH_2Cl_2	0.9 : 1.0	65[h]

[a]Reagents and conditions: **2** (0.05 mmol), Lewis acid (1.5 equiv), reducing agent (1.5 equiv), solvent (0.5 mL), -78 °C, 15 min. [b]Determined by ^1H NMR. [c]Isolated yield of **1** and **1'**. [d]$NaBH_4$ (4.0 equiv), MeOH (0.35 mL), 0 °C, 1 h. [e]DIBALH (1.0 equiv), toluene (0.15 mL), -78 °C, 1 h. [f]$Zn(BH_4)_2$ (1.0 equiv), CH_2Cl_2 (0.15 mL), 0 °C to rt, 1 h. [g]py=pyridine, [h] 3 h.

The procedure to synthesis of *N,N*-diethyl-4-hydroxy-2-(trimethylsilyl)benzofuran-5-carboxamide lead to opening furan ring

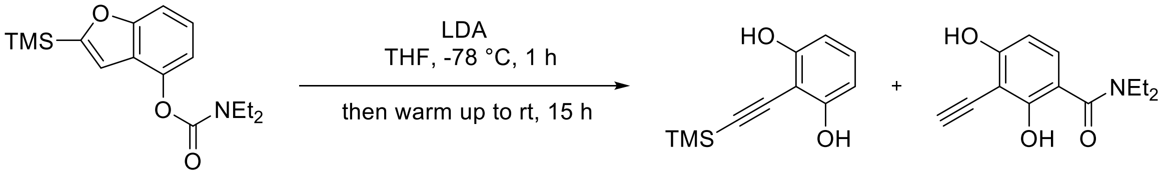

16

To a solution of 2-(trimethylsilyl)benzofuran-4-yl diethylcarbamate **16** (36.6 mg, 0.12 mmol) in dry THF (0.36 mL) was cooled at -78 °C. Then, LDA (1.0 M in THF, 0.42 mL, 0.42 mmol) was added to the solution dropwise and stirred at -78 °C for 1 hour. Then, the mixture was warmed up to rt and stirred for 15 h. Upon completion, saturated NH_4Cl (0.5 mL) was added. Then, the mixture was acidified by 1 M HCl to pH 3 and extracted with EtOAc (3×5 mL). The combined organic layers were dried over anhydrous Na_2SO_4, filtrated, and concentrated *in vacuo*. The crude product was purified by column chromatography (silica gel, 5 to 30% EtOAc/Hexane) to afford the ring opening products.

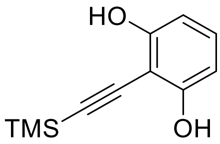

2-((trimethylsilyl)ethynyl)benzene-1,3-diol

^{1}H NMR (500 MHz, $CDCl_3$) δ: 7.12 (t, *J* = 8.2 Hz, 1H), 6.50 (d, *J* = 8.2 Hz, 2H), 5.55 (s, 2H), 0.30 (s, 9H); HRMS (EI) m/z: $[M]^+$ Calcd for $C_{11}H_{14}O_2Si$ 206.0758; Found 206.0764.

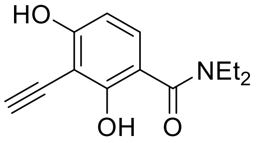

***N,N*-diethyl-3-ethynyl-2,4-dihydroxybenzamide**

^{1}H NMR (500 MHz, $CDCl_3$) δ: 11.33 (s, 1H), 7.23 (d, *J* = 8.7 Hz, 1H), 6.47 (d, *J* = 8.7 Hz, 1H), 6.16 (br s, 1H), 3.73 (s, 1H), 3.50 (q, *J* = 7.1 Hz, 4H), 1.27 (t, *J* = 7.1 Hz, 6H); $^{13}C\{^1H\}$ NMR (125 MHz, $CDCl_3$) δ: 171.4, 162.6, 160.7, 129.7, 110.0, 105.2, 98.8, 89.0,74.4, 42.5, 13.5; HRMS (ESI) m/z: $[M+H]^+$ Calcd for $C_{13}H_{16}NO_3$ 234.1125; Found 234.1122.

3. ^{1}H and $^{13}C\{^{1}H\}$ Spectra of Products

2-Iodobenzene-1,3-diol (7)

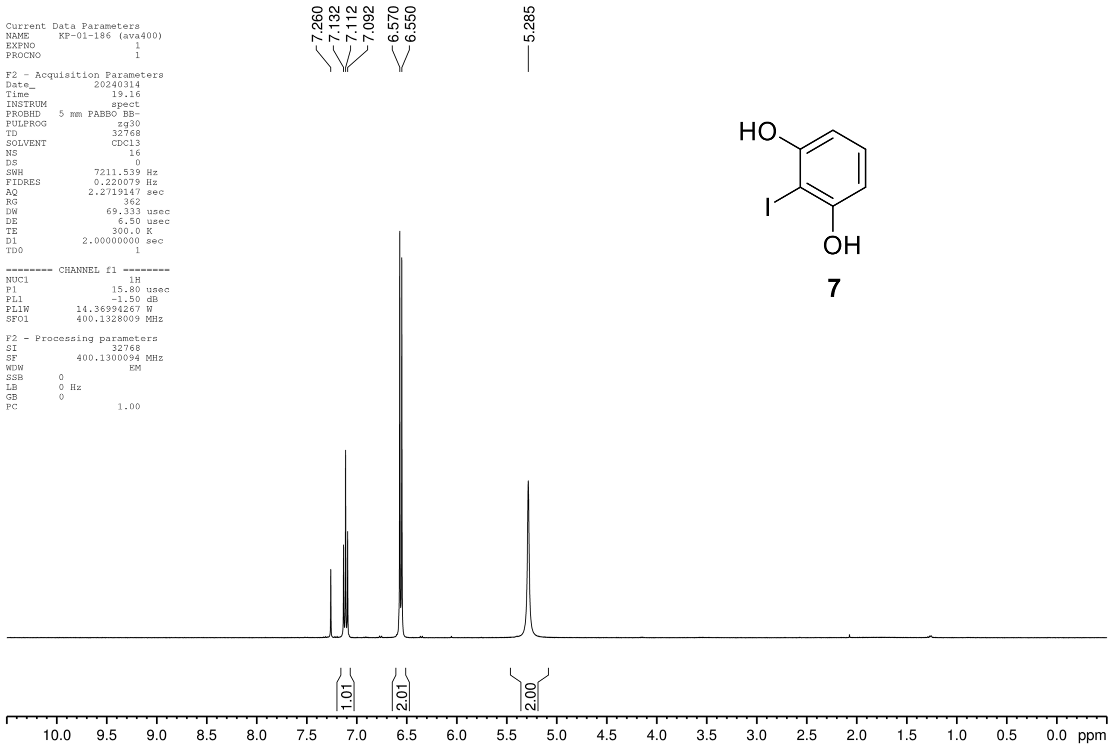

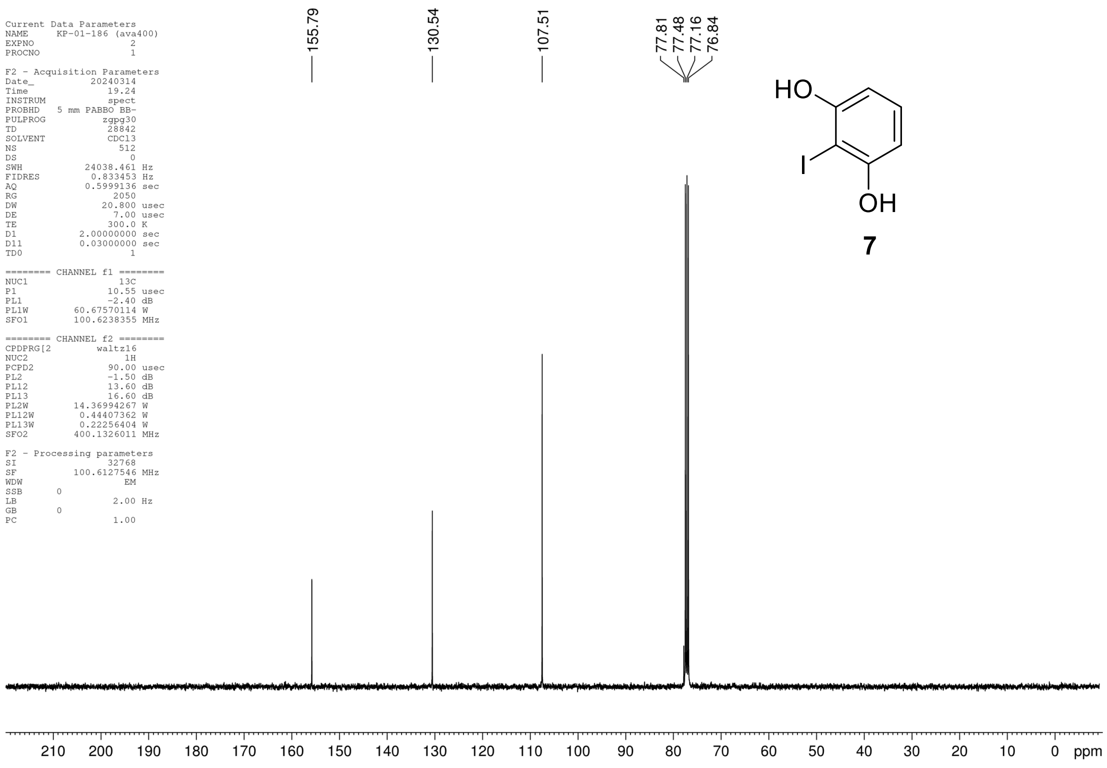

2-((triisopropylsilyl)ethynyl)benzene-1,3-diol (8)

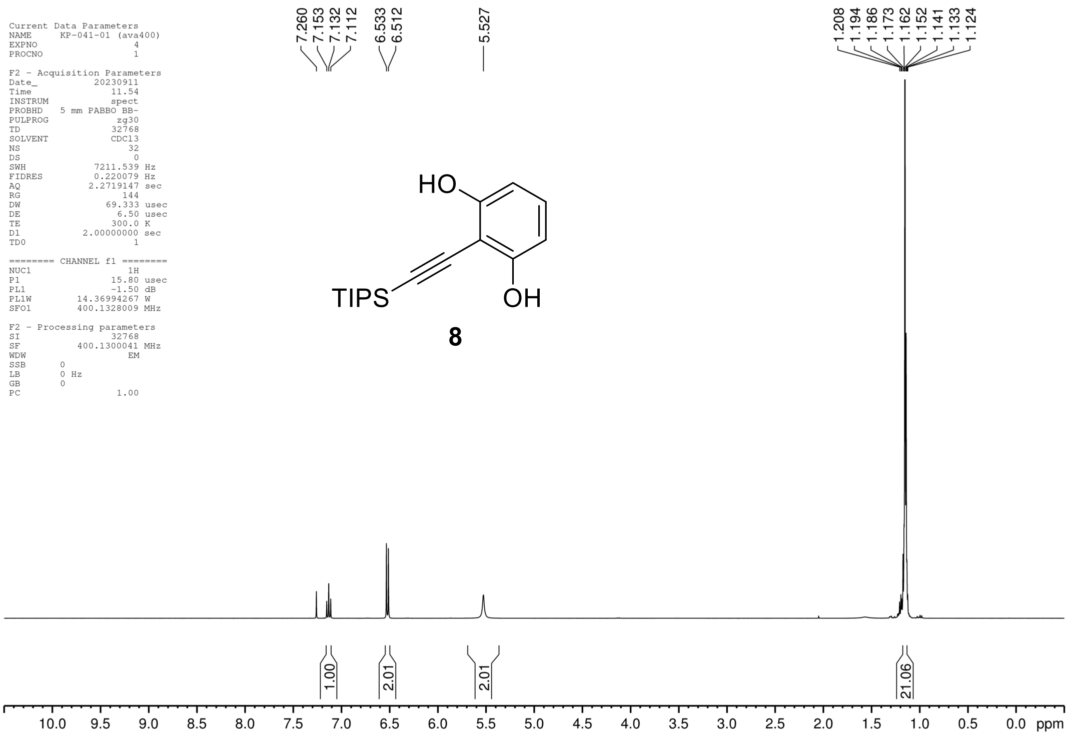

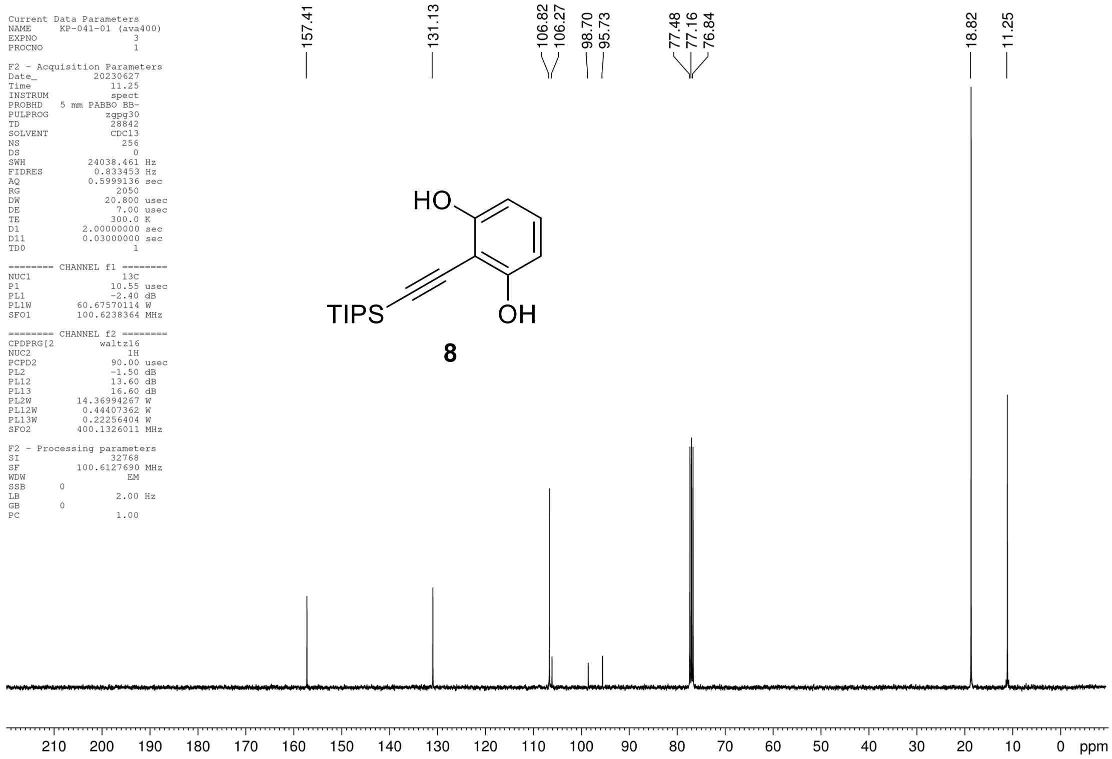

2-(triisopropylsilyl)benzofuran-4-ol (9)

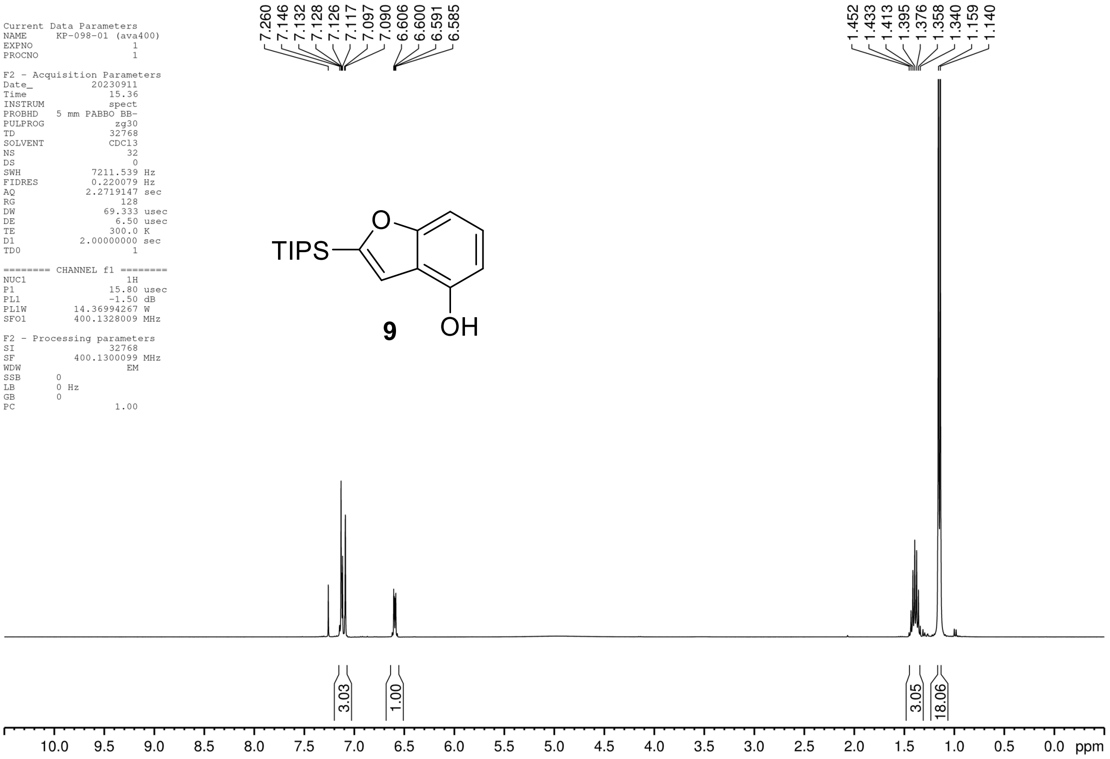

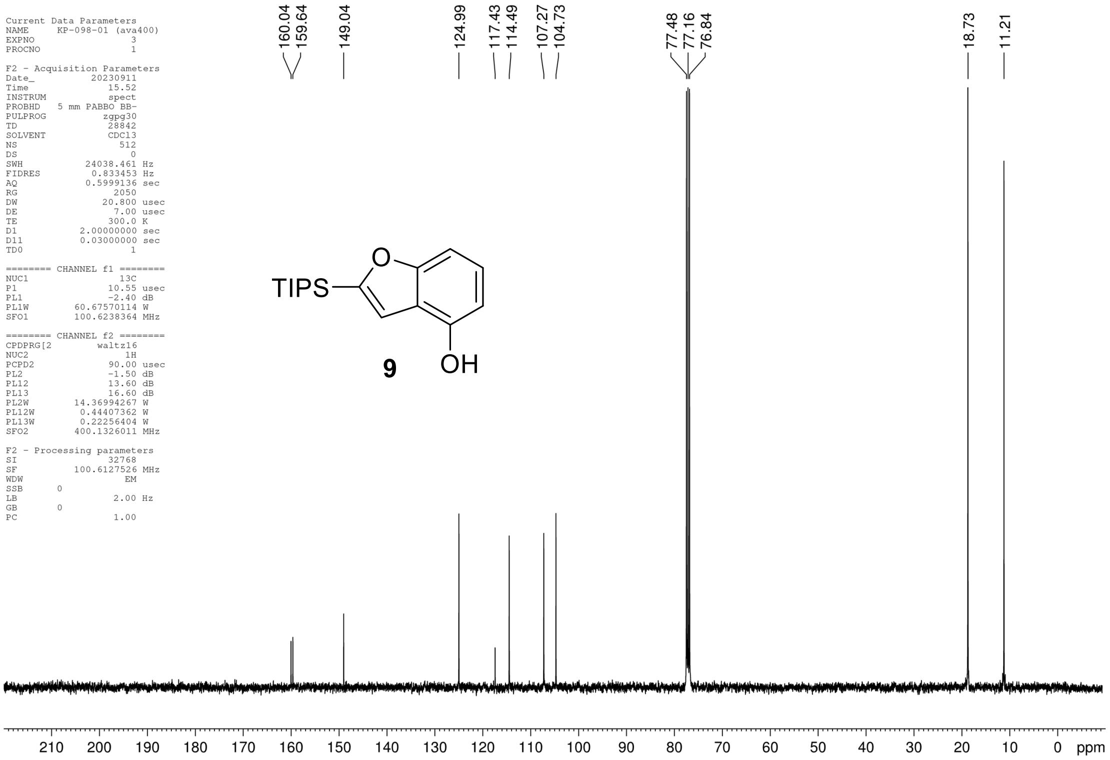

2-(triisopropylsilyl)benzofuran-4-yl diethylcarbamate (10)

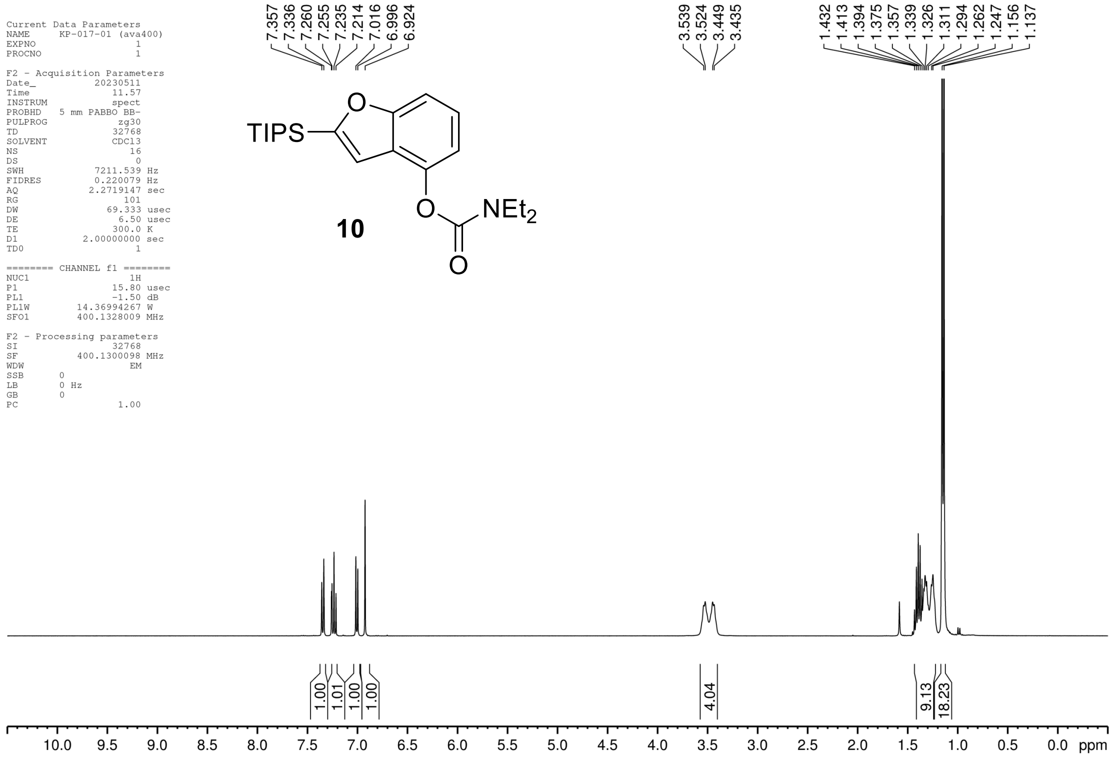

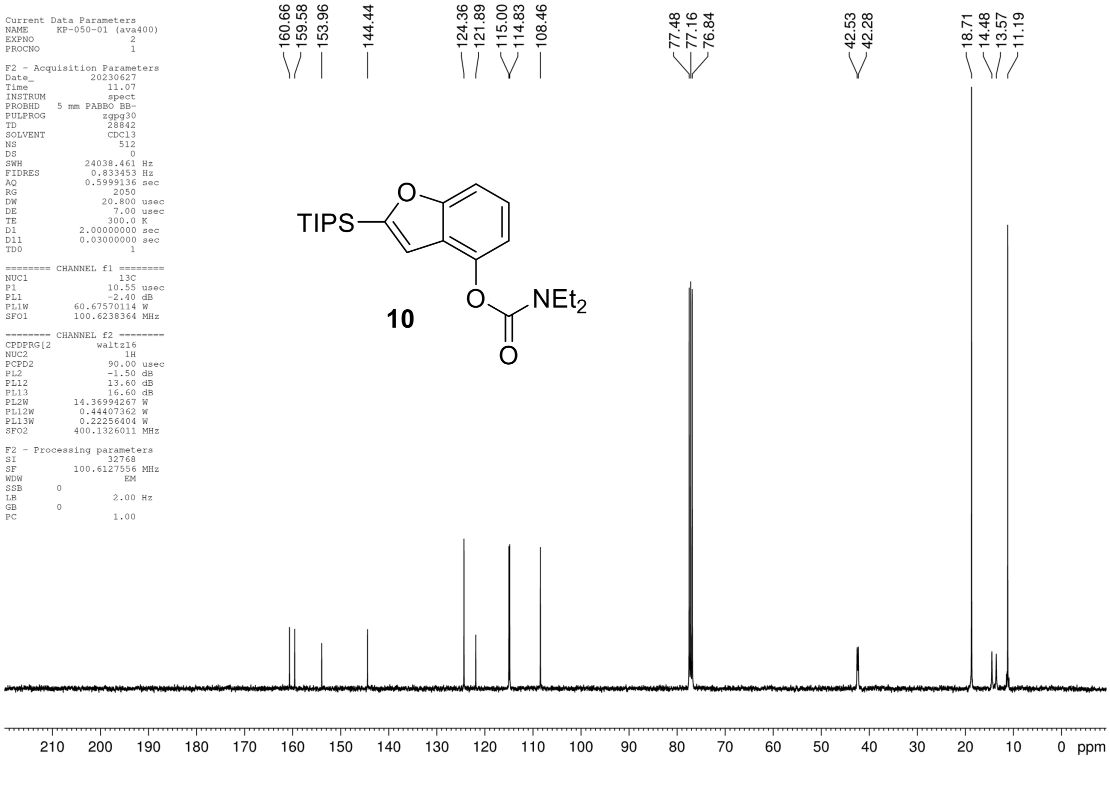

N,N-diethyl-4-hydroxy-2-(triisopropylsilyl)benzofuran-5-carboxamide (11)

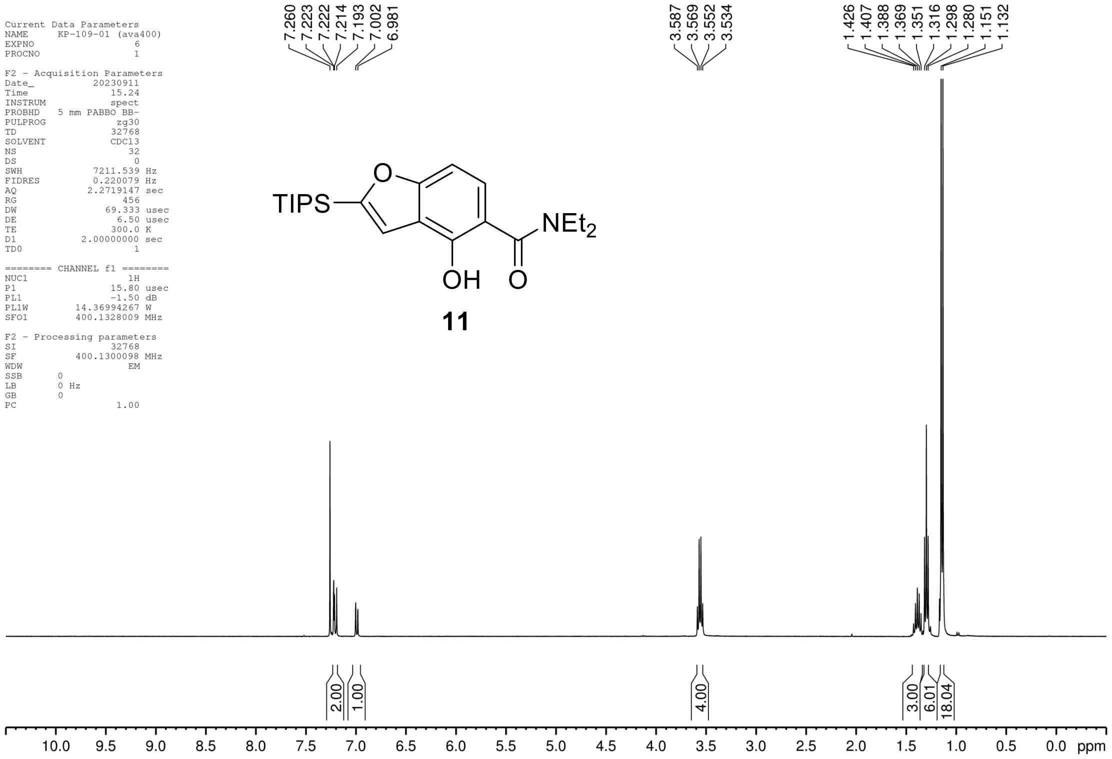

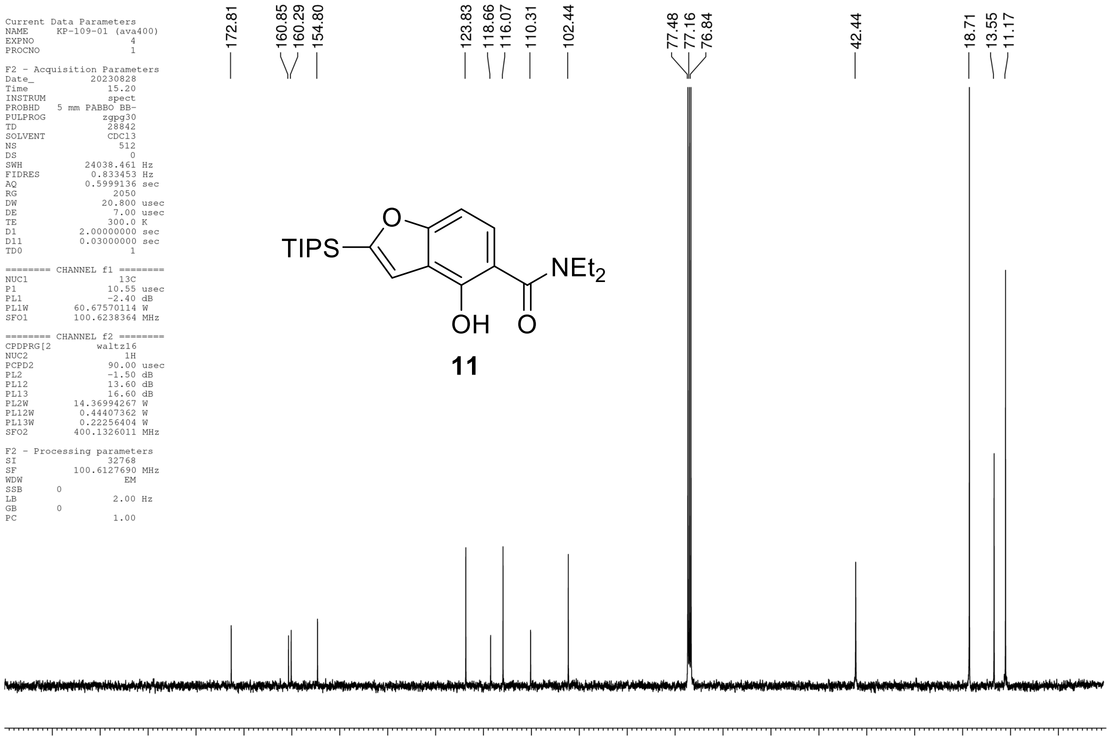

N,N-diethyl-4-methoxy-2-(triisopropylsilyl)benzofuran-5-carboxamide (12)

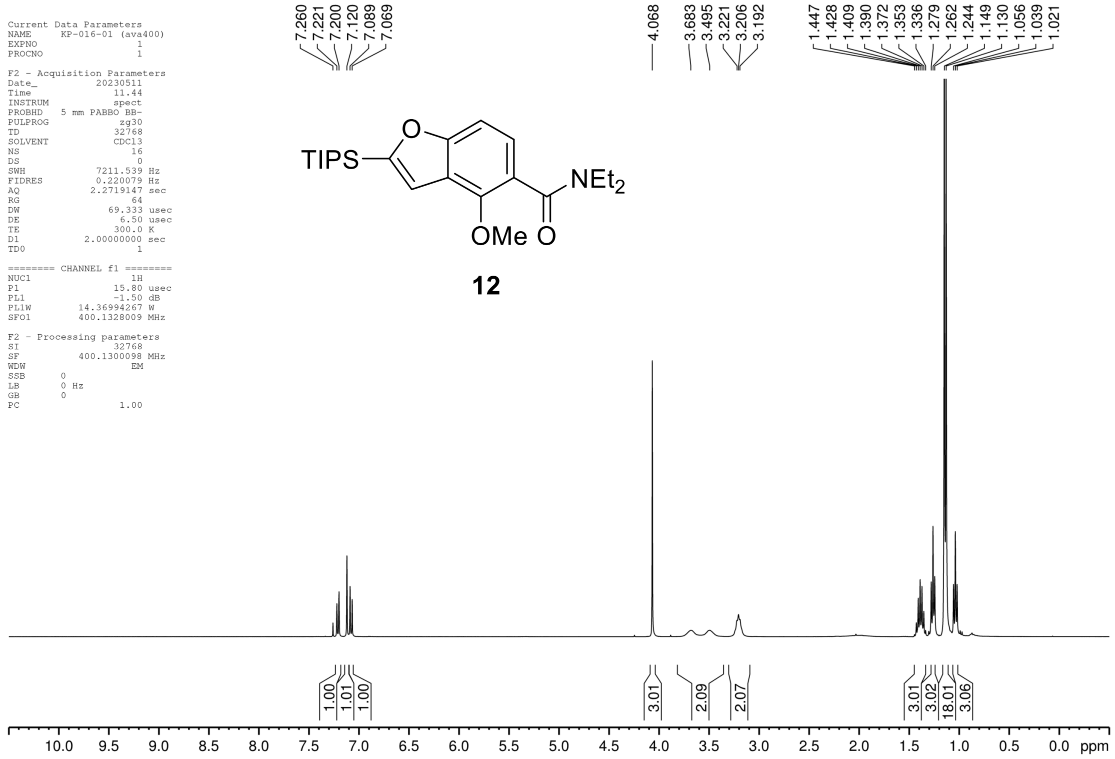

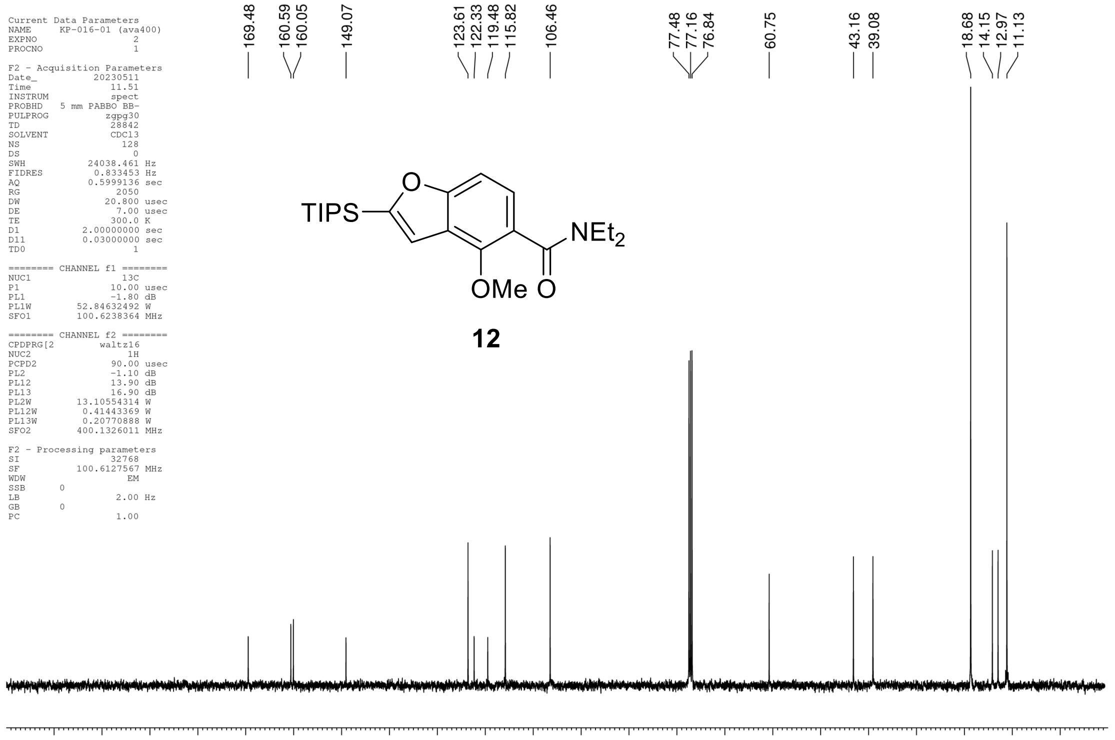

(*R*)-2-((*tert*-butyldimethylsilyl)oxy)-*N*-methoxy-*N*-methylpropanamide ((*R*)-3)

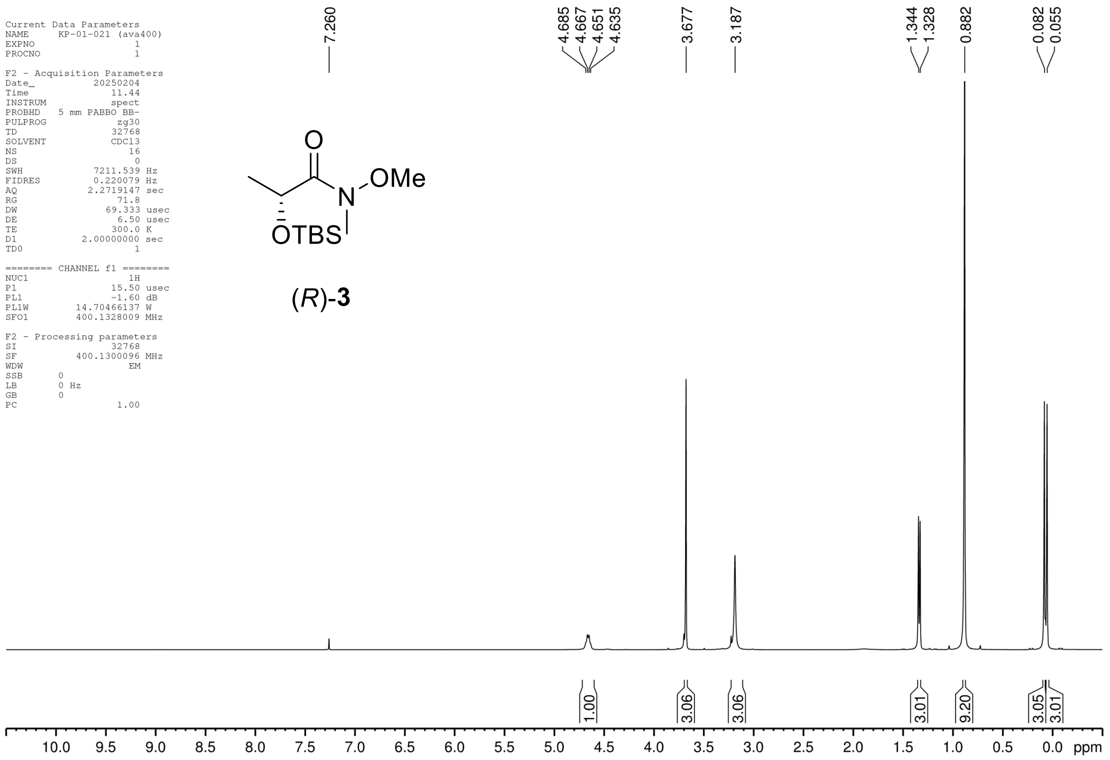

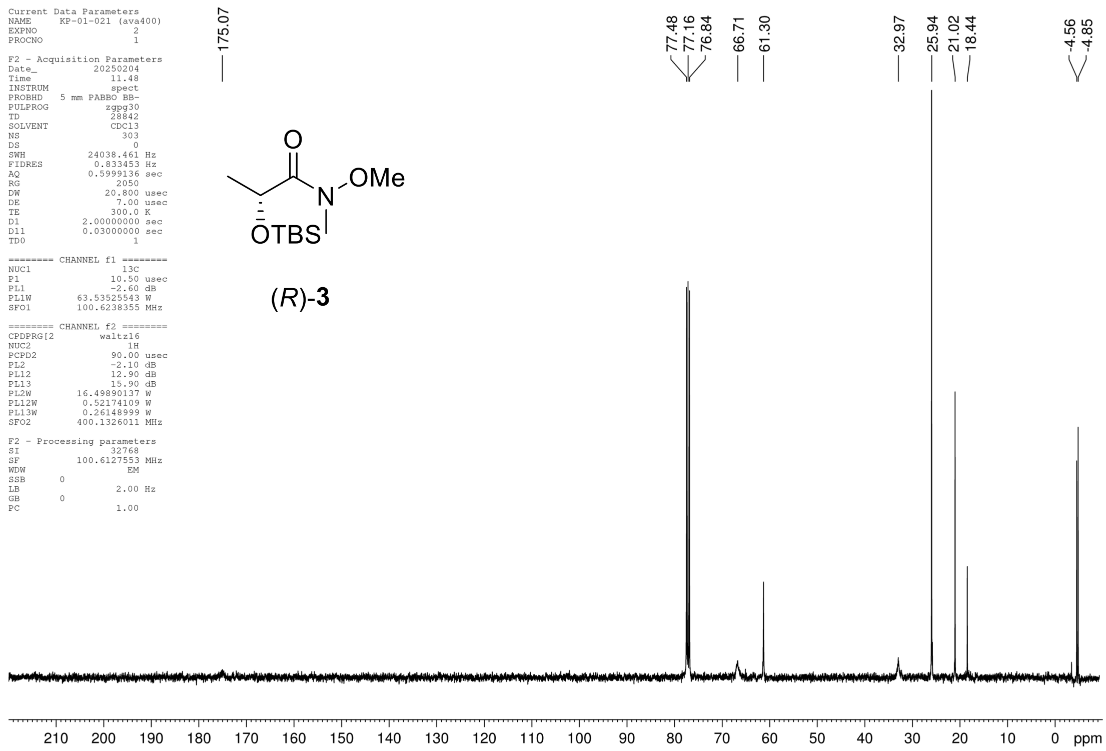

(*R*)-6-(2-((*tert*-butyldimethylsilyl)oxy)propanoyl)-*N*,*N*-diethyl-4-methoxy-2-(triisopropylsilyl)benzofuran-5-carboxamide (13)

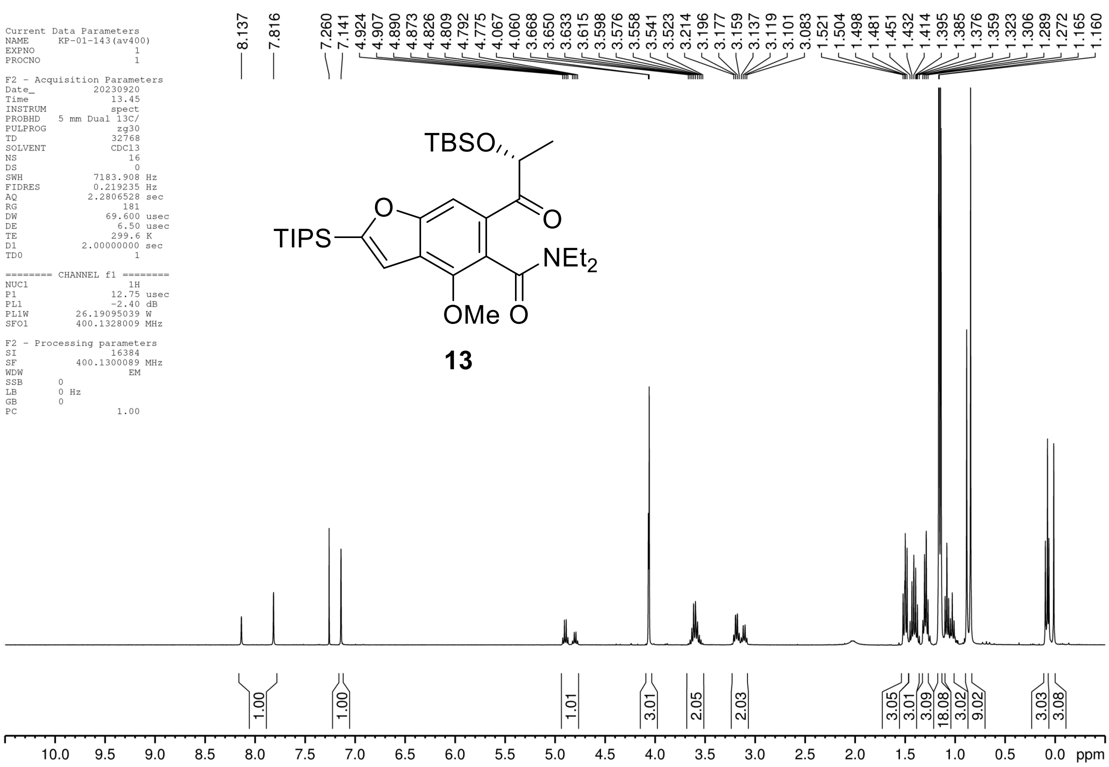

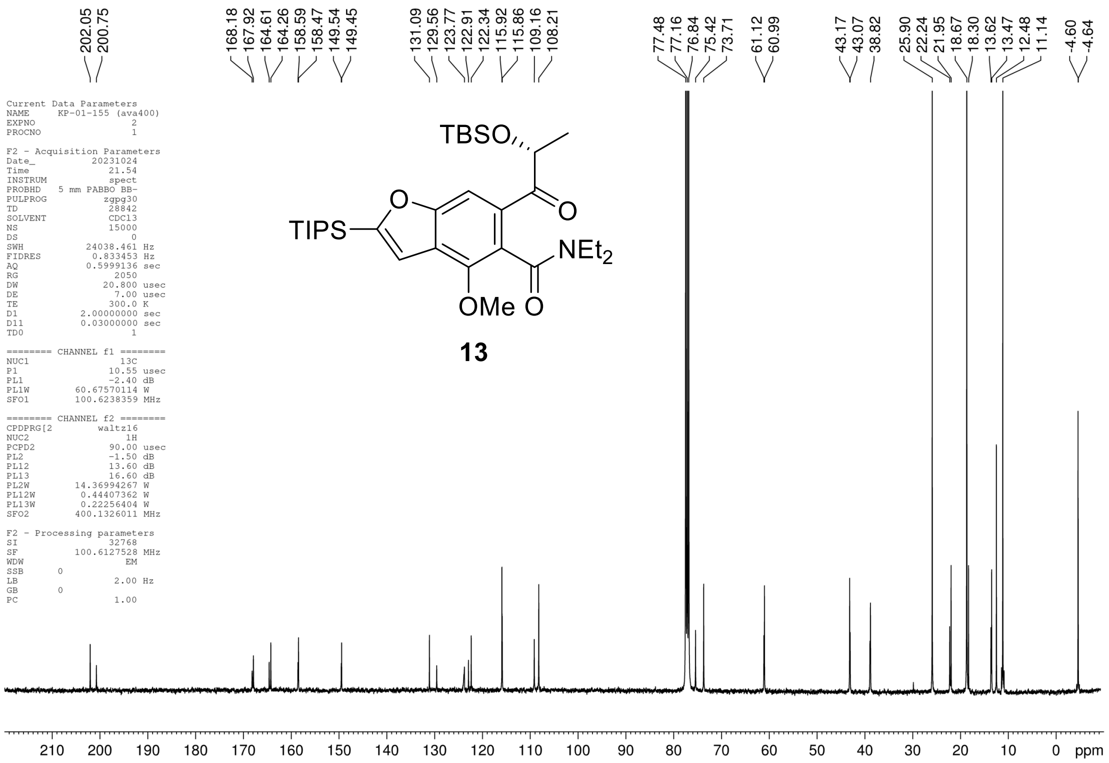

4-(*tert*-butyl)-*N*,*N*-diethyl-2-(triisopropylsilyl)benzofuran-5-carboxamide (14)

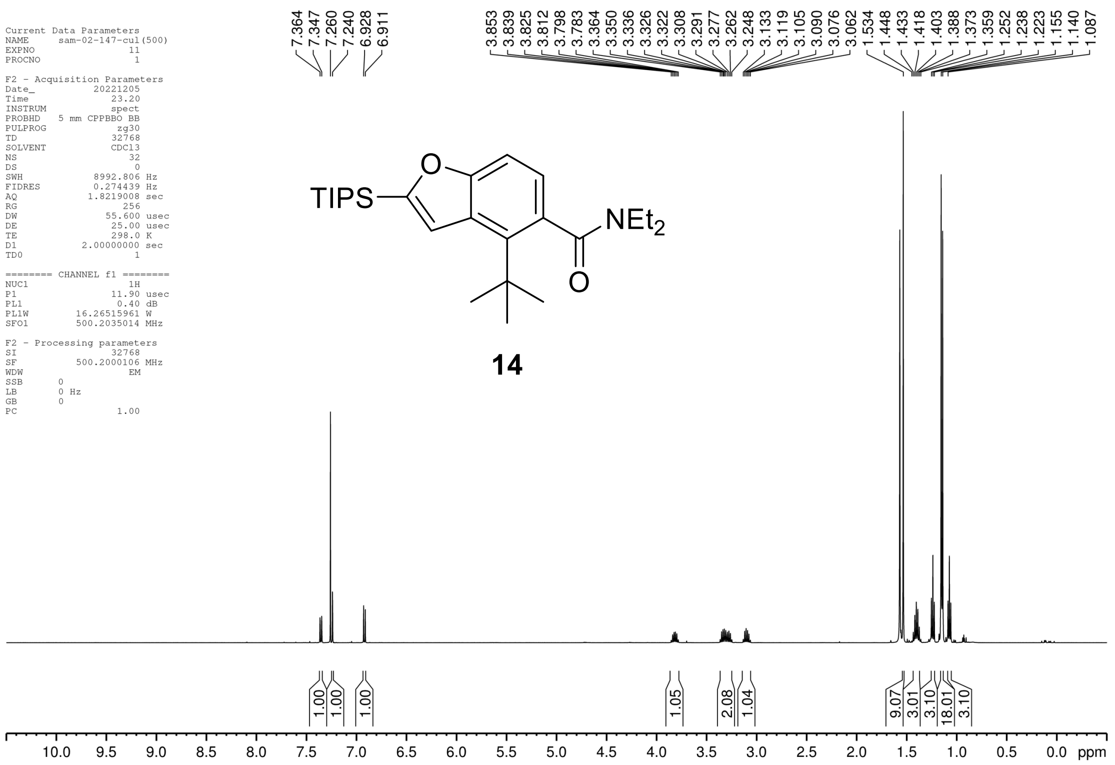

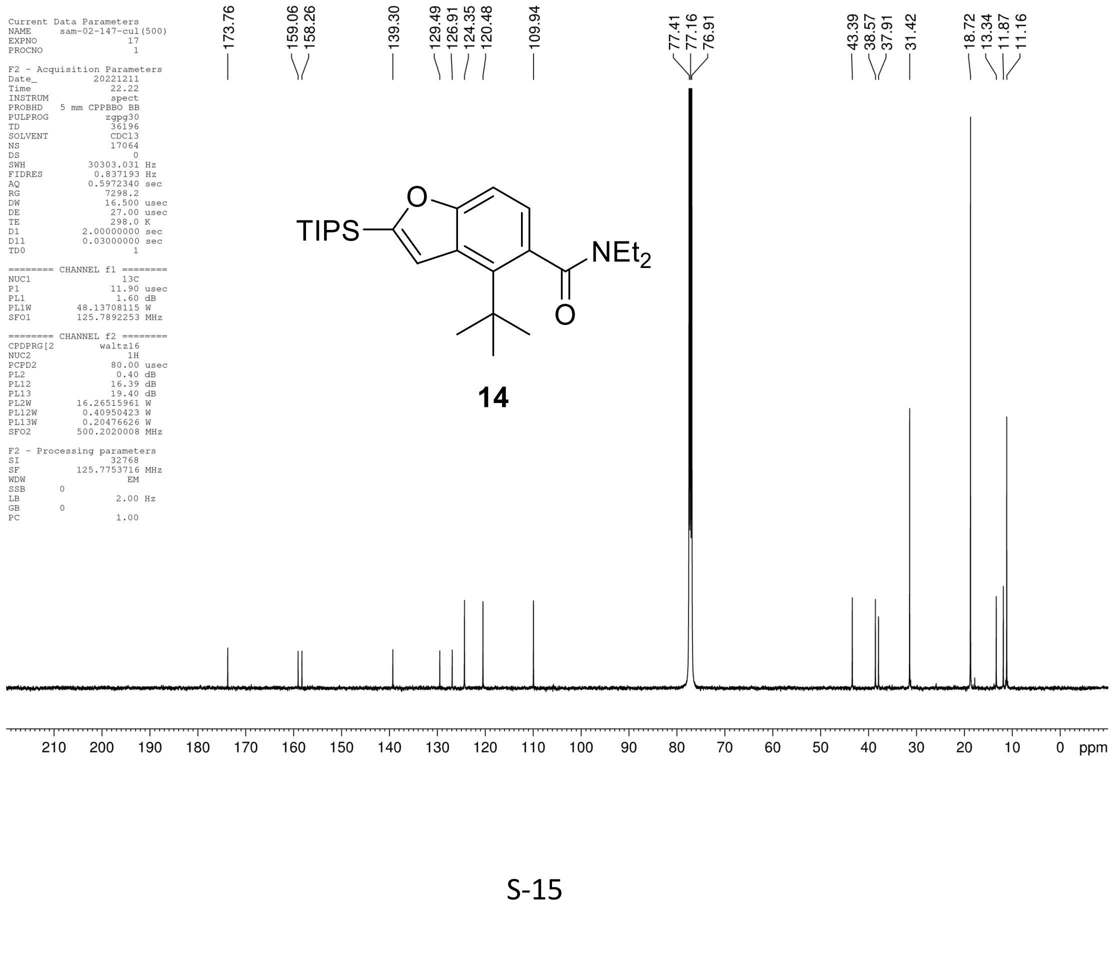

(*R*)-*N,N*-diethyl-6-(2-hydroxypropanoyl)-4-methoxybenzofuran-5-carboxamide (2)

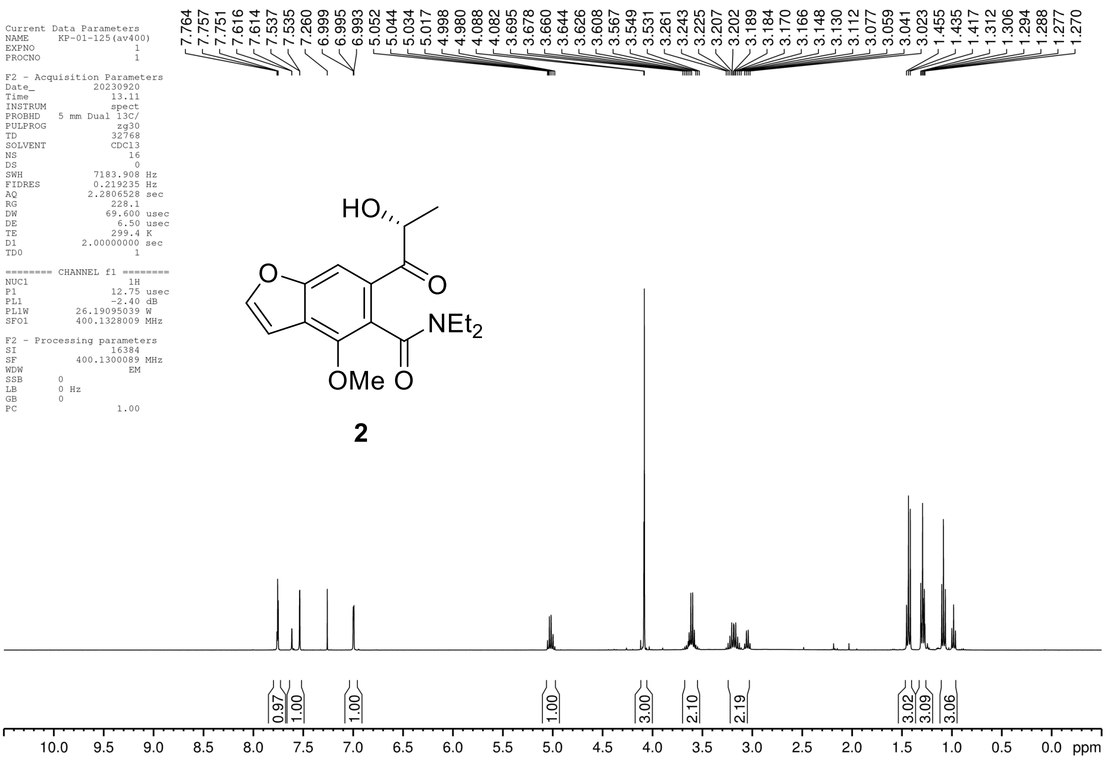

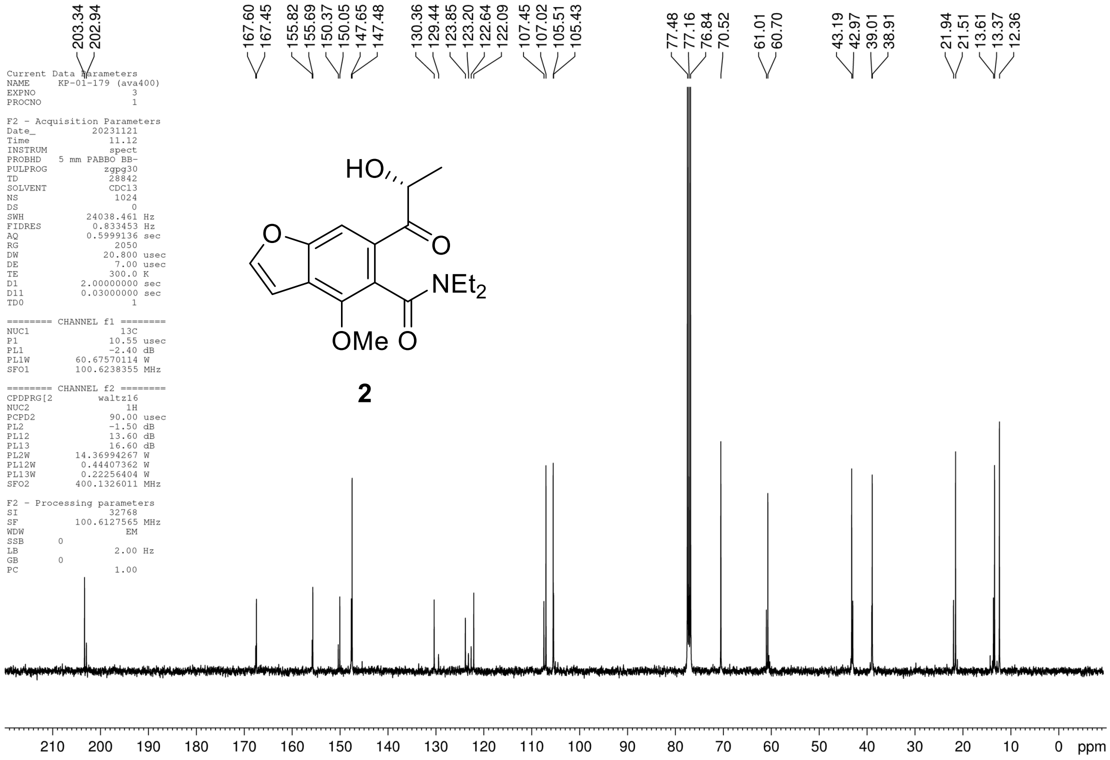

(*S*)-7-((*R*)-1-hydroxyethyl)-4-methoxybenzo[1,2-*b*:4,5-*c'*]difuran-5(7*H*)-one (1)

(+)-nervione

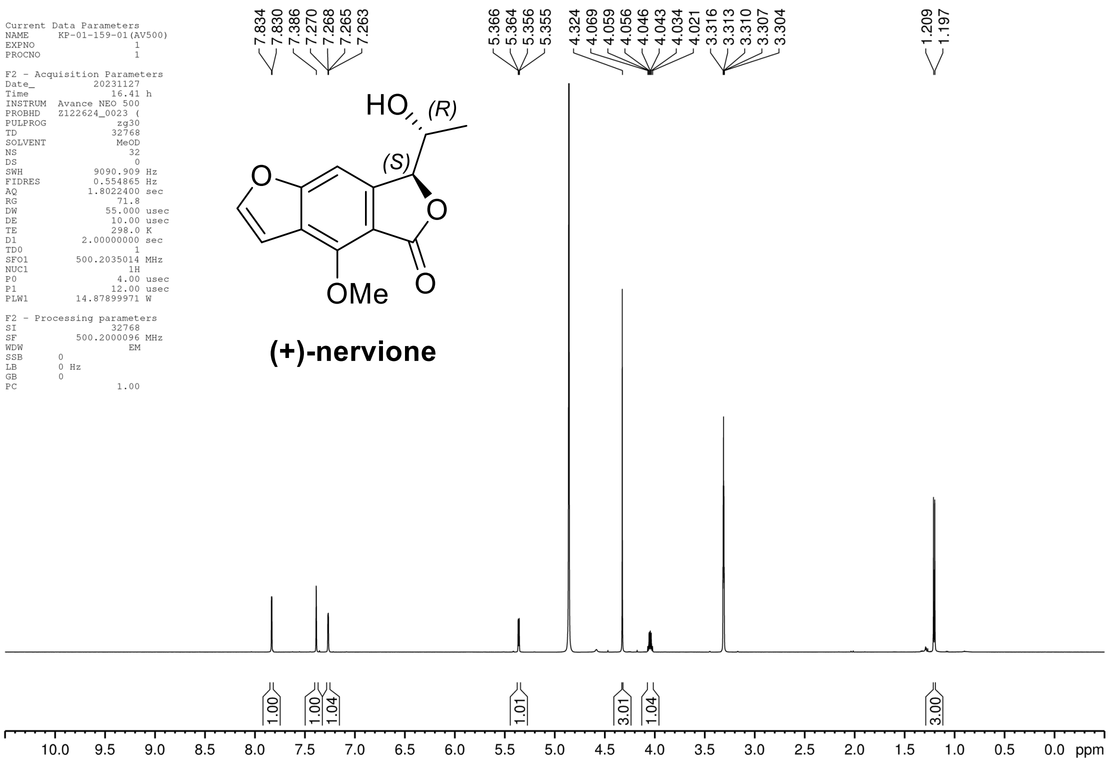

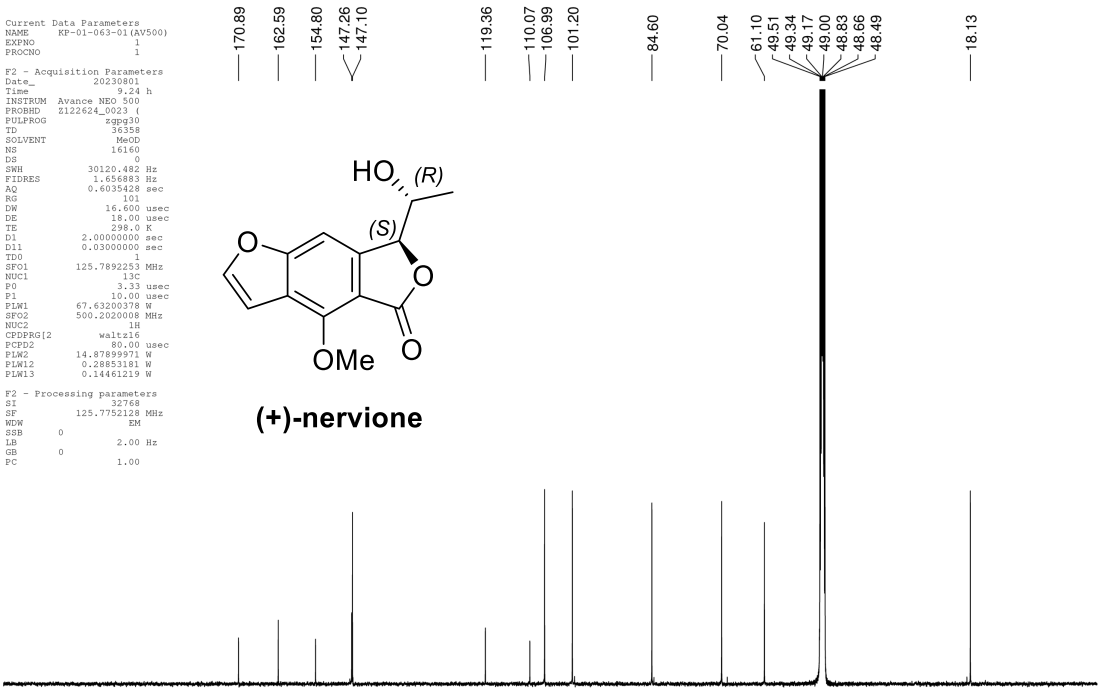

(*R*)-7-((*S*)-1-hydroxyethyl)-4-methoxybenzo[1,2-*b*:4,5-*c'*]difuran-5(*7H*)-one (−)-nervione

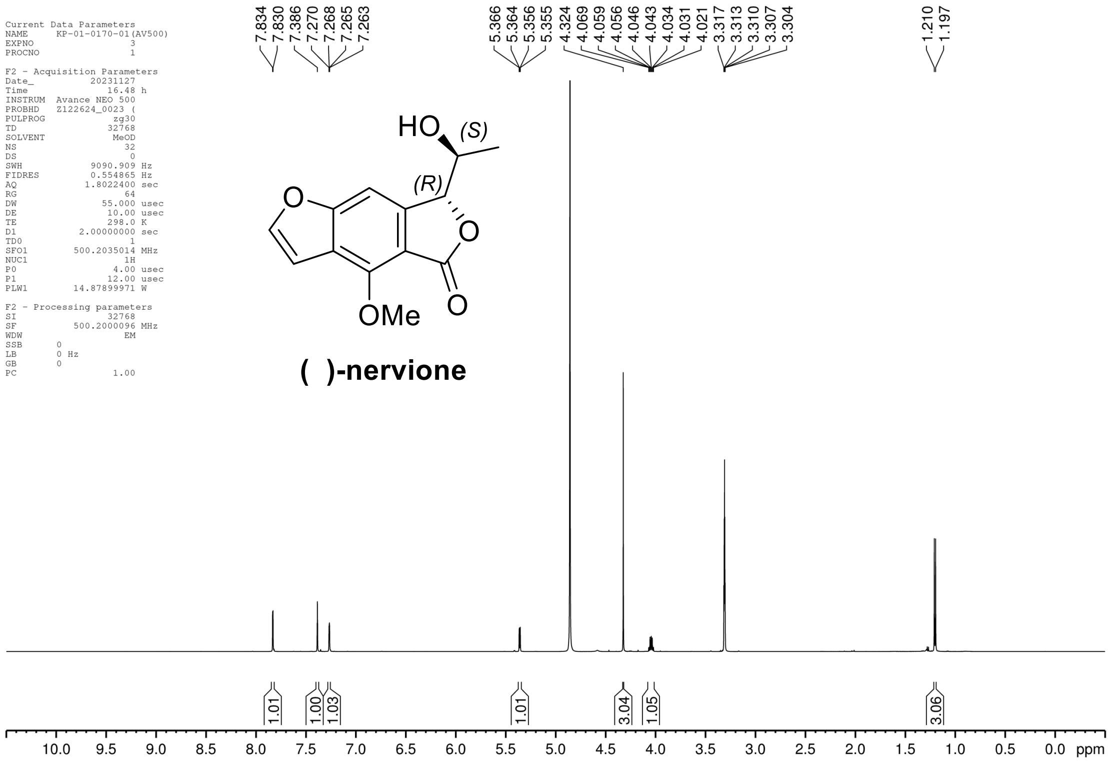

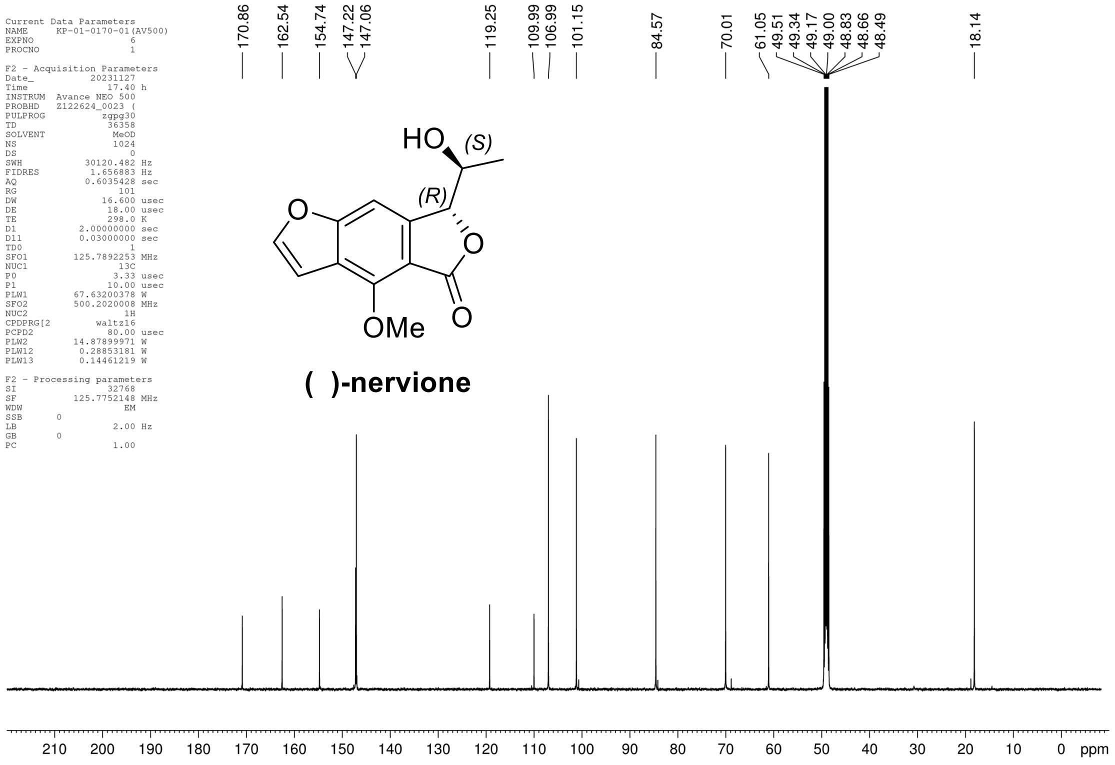

2-(trimethylsilyl)benzofuran-4-yl diethylcarbamate (16)

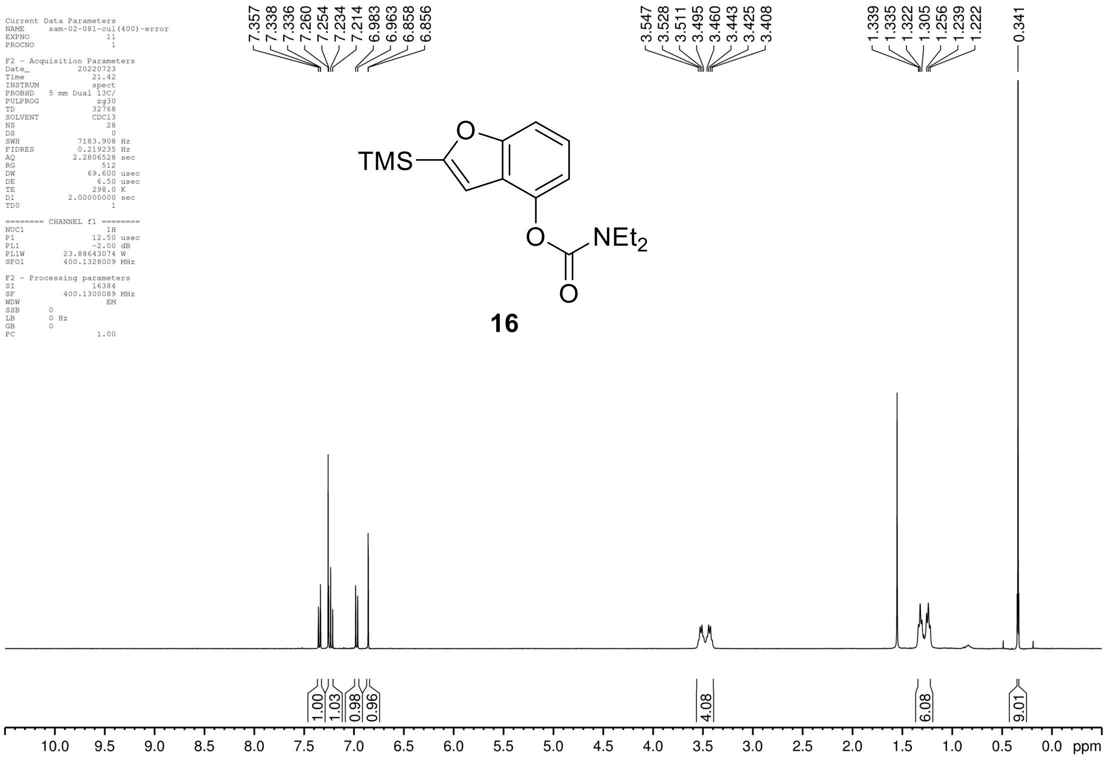

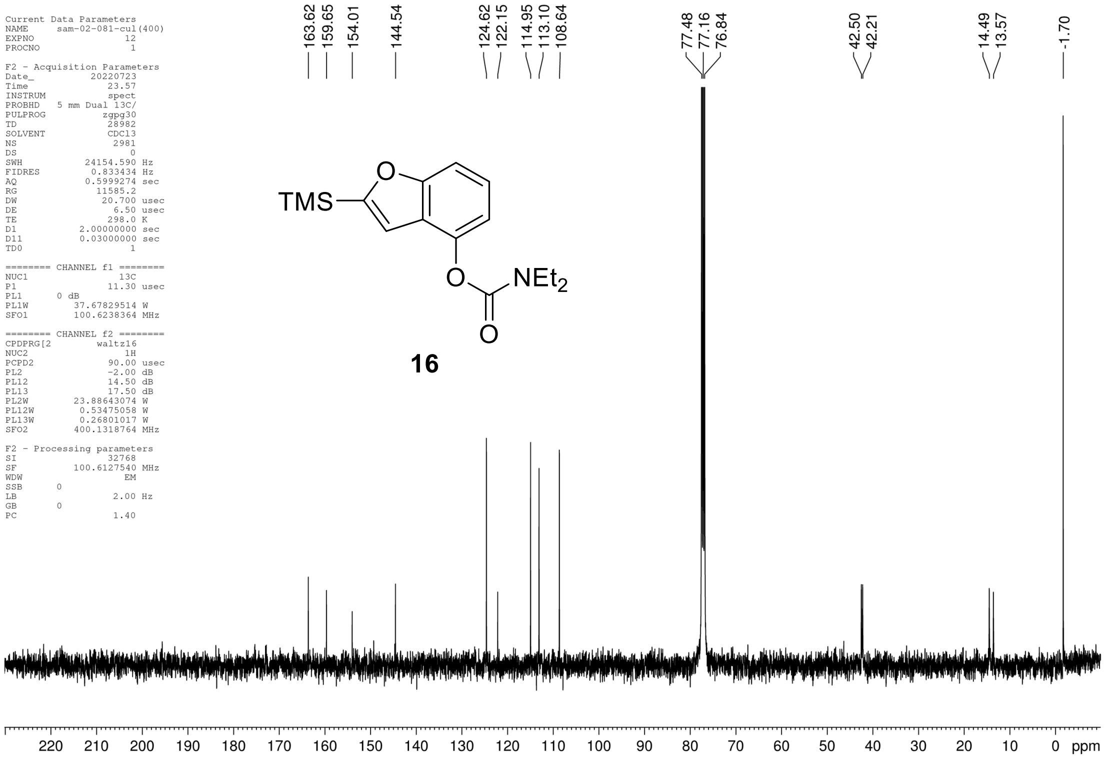

2-((trimethylsilyl)ethynyl)benzene-1,3-diol

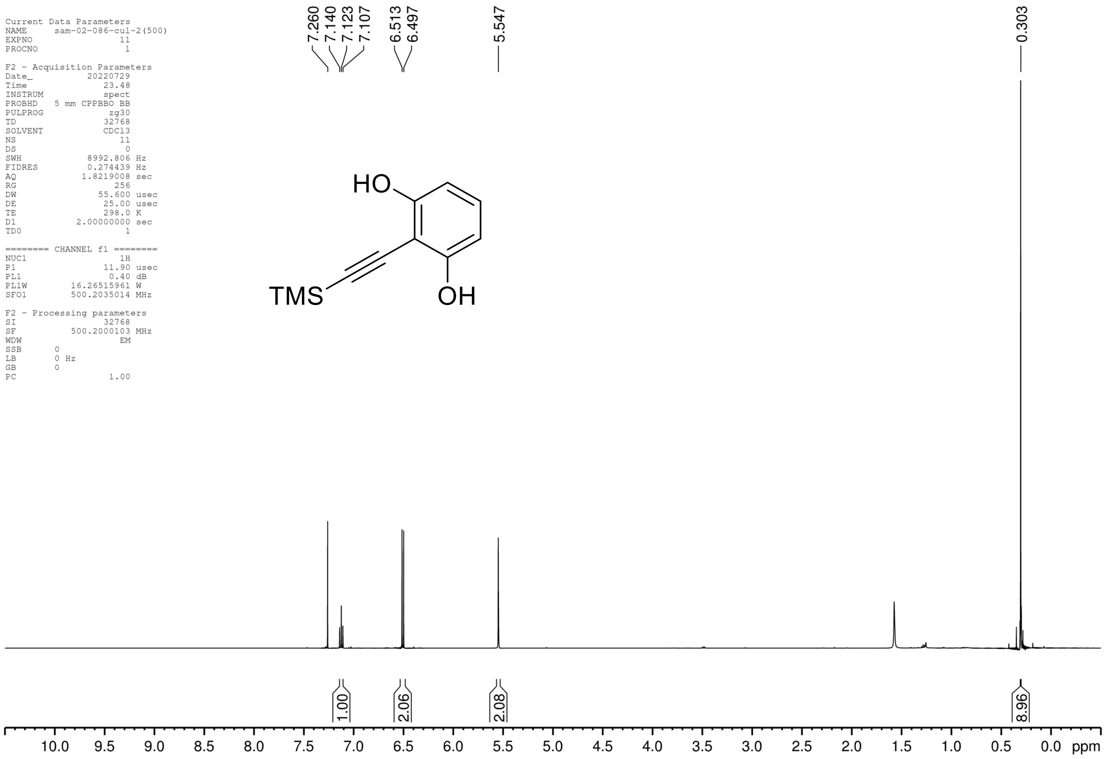

***N,N*-diethyl-3-ethynyl-2,4-dihydroxybenzamide**

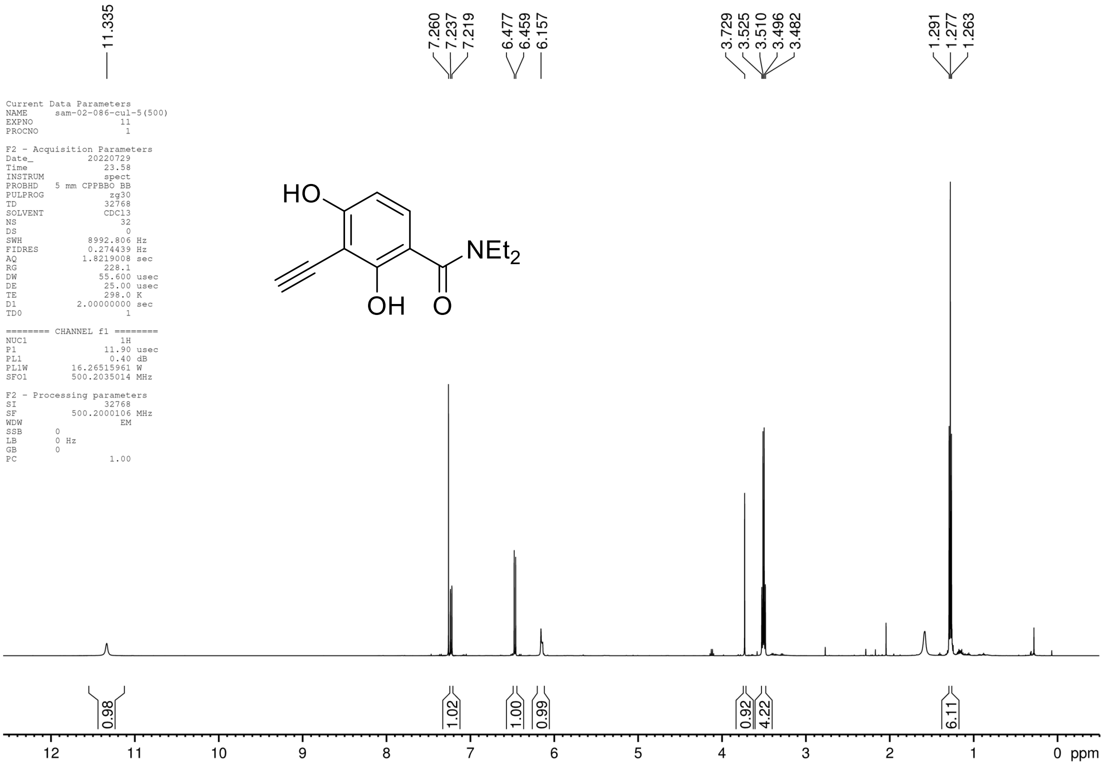

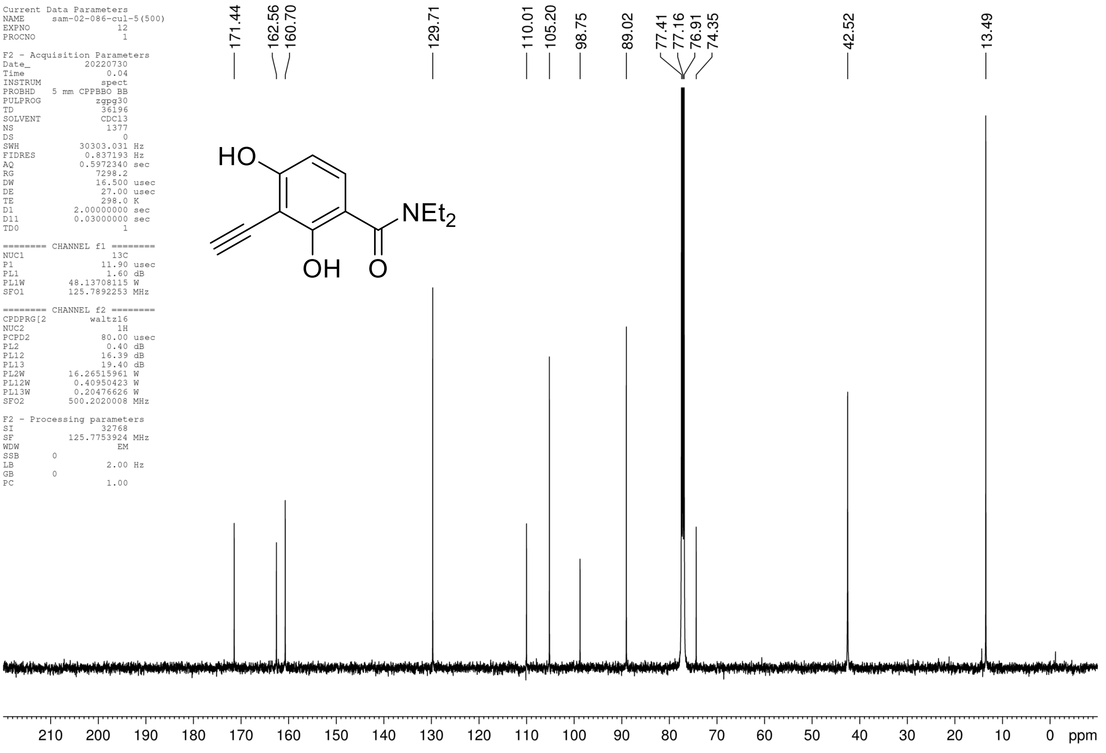

(*R*)-2-((*tert*-butyldimethylsilyl)oxy)propanal ((*R*)-17)

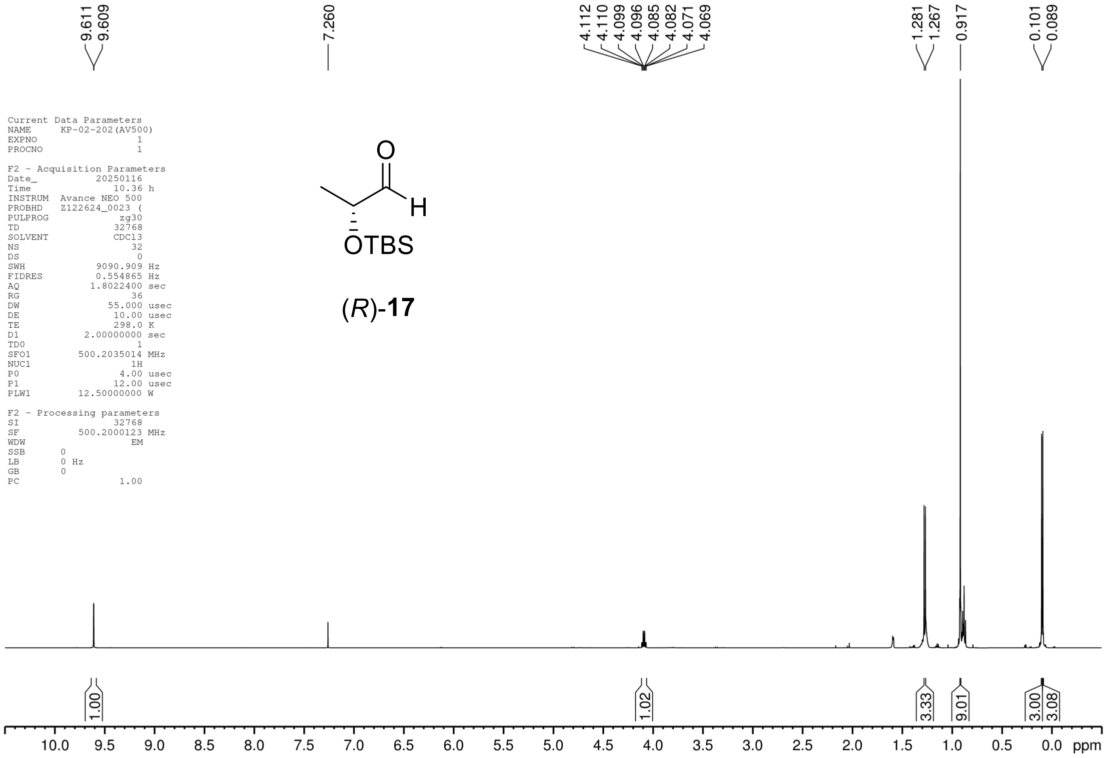

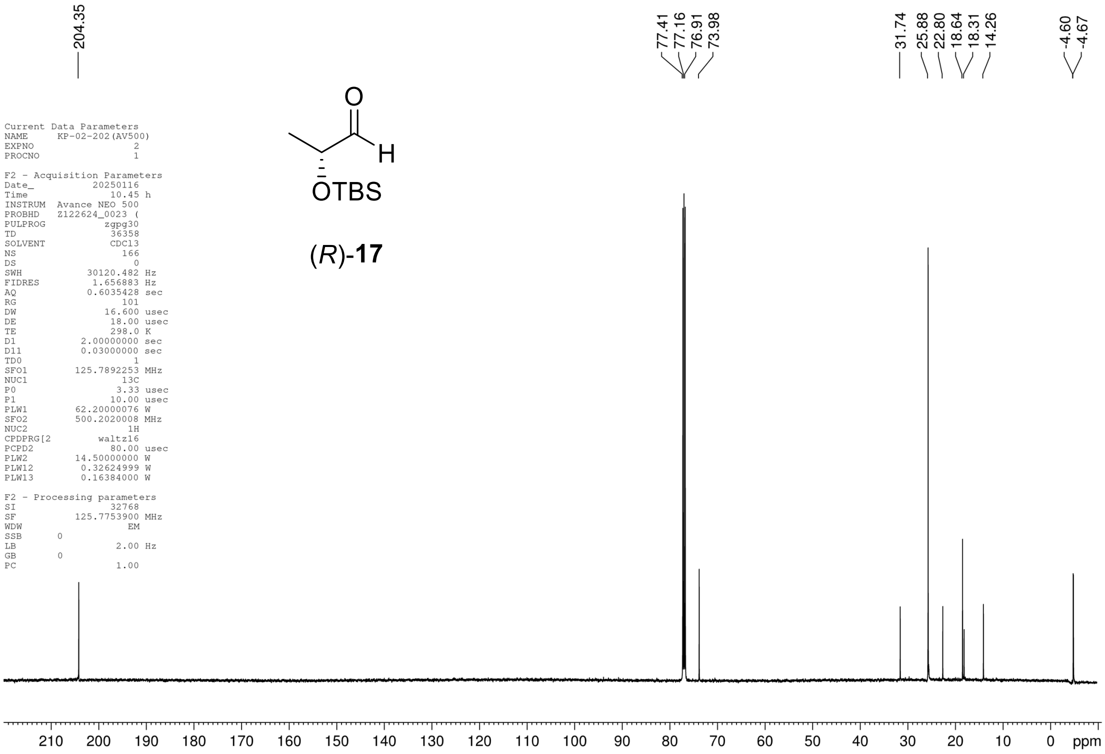

4. Circular Dichroism

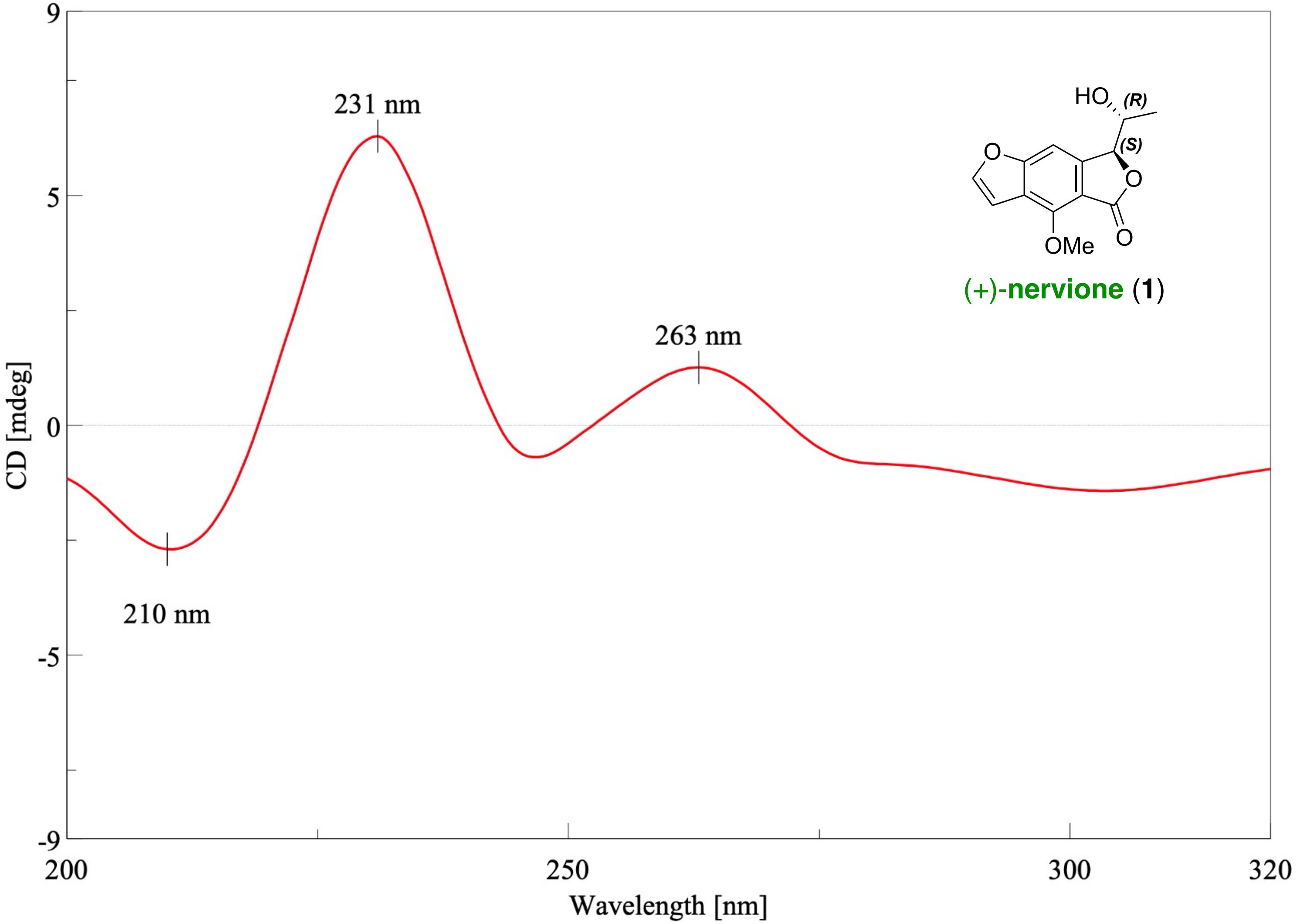

Figure S1. CD spectrum of (*S*)-7-((*R*)-1-hydroxyethyl)-4-methoxybenzo[1,2-*b*:4,5-*c'*]difuran-5(*7H*)-one (0.1 M in MeOH)

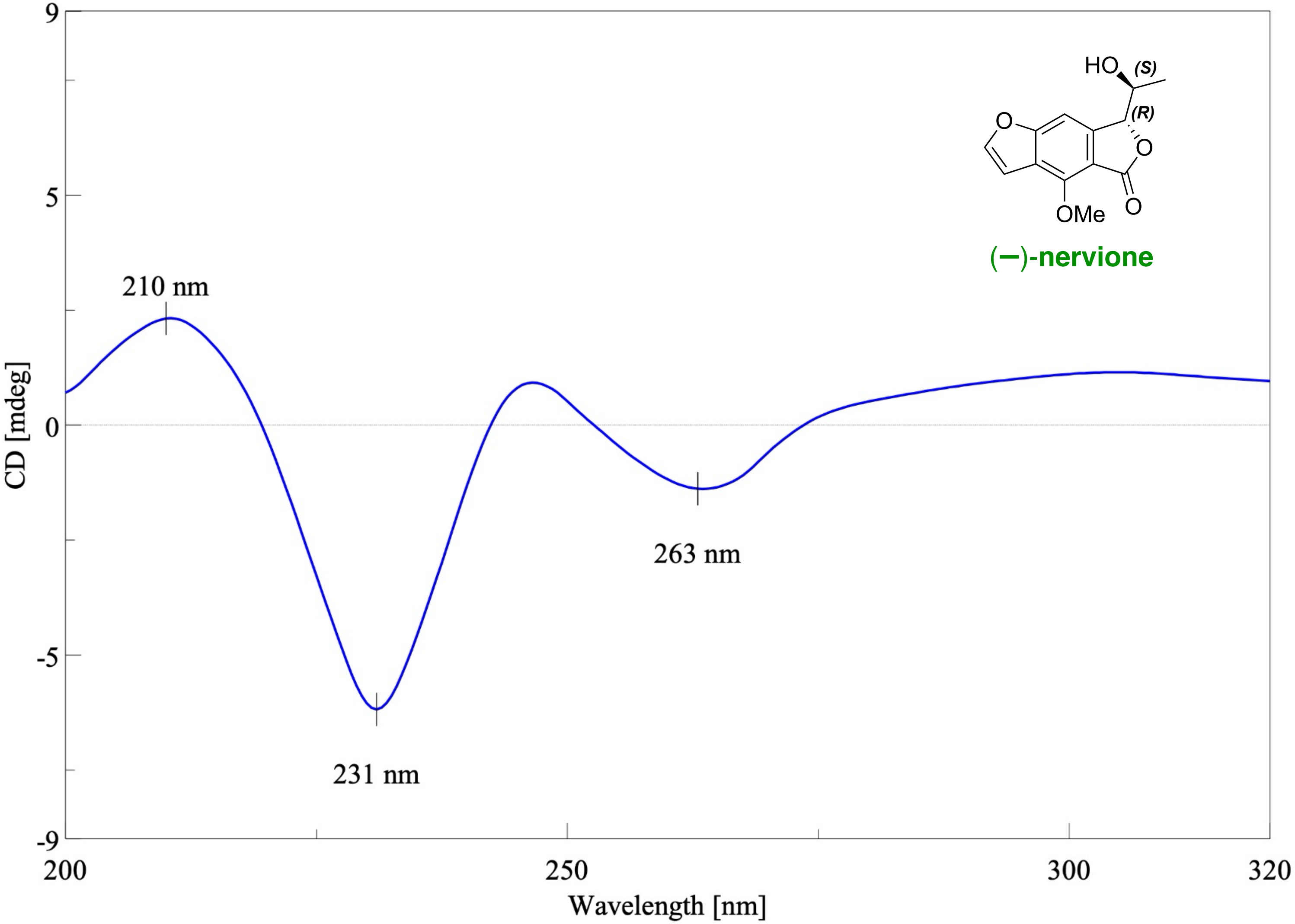

Figure S2. CD spectrum of (*R*)-7-((*S*)-1-hydroxyethyl)-4-methoxybenzo[1,2-*b*:4,5-*c'*]difuran-5(7*H*)-one (0.1 M in MeOH)

5. X-Ray Crystal Structure Analysis

X-ray diffraction data of compound 1 (+)-nervione

(Displacement ellipsoids are drawn at the 50% probability level, CCDC-2340879)

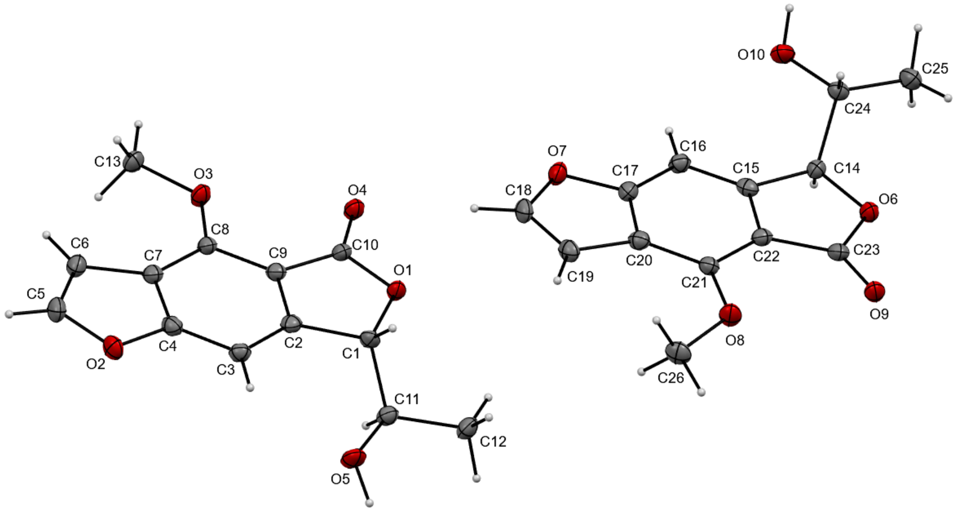

Table S3. Crystal data and structure refinement for i19180.

Identification code	i19180	
Empirical formula	C13 H12 O5	
Formula weight	248.23	
Temperature	100.0(2) K	
Wavelength	1.54178 Å	
Crystal system	Triclinic	
Space group	P 1	
Unit cell dimensions	a = 7.7527(2) Å	α= 89.8310(10)°.
	b = 8.0743(2) Å	β= 87.5890(10)°.
	c = 8.8740(2) Å	γ = 88.4040(10)°.
Volume	554.78(2) Å^3	
Z	2	
Density (calculated)	1.486 Mg/m^3	
Absorption coefficient	0.972 mm^{-1}	
F(000)	260	
Crystal size	0.103 x 0.066 x 0.060 mm^3	
Theta range for data collection	4.988 to 72.603°.	
Index ranges	-9<=h<=8, -9<=k<=9, -10<=l<=10	
Reflections collected	15323	
Independent reflections	3967 [R(int) = 0.0406]	
Completeness to theta = 67.679°	98.6 %	

Absorption correction	Numerical
Max. and min. transmission	1 and 0.762
Refinement method	Full-matrix least-squares on F^2
Data / restraints / parameters	3967 / 3 / 337
Goodness-of-fit on F^2	1.091
Final R indices [I>2sigma(I)]	R1 = 0.0314, wR2 = 0.0902
R indices (all data)	R1 = 0.0322, wR2 = 0.0906
Absolute structure parameter	0.04(6)
Extinction coefficient	n/a
Largest diff. peak and hole	0.256 and -0.300 e.Å^{-3}

Table S4. Atomic coordinates (x 10^4) and equivalent isotropic displacement parameters ($Å^2$x 10^3) for i19180. U(eq) is defined as one third of the trace of the orthogonalized U^{ij} tensor.

	x	y	z	U(eq)
O(1)	5883(2)	4047(2)	2833(2)	18(1)
O(2)	8324(2)	2585(2)	-3448(2)	21(1)
O(3)	8548(2)	7218(2)	-188(2)	20(1)
O(4)	6891(2)	6611(2)	2768(2)	20(1)
O(5)	6836(3)	-135(2)	1529(2)	25(1)
O(6)	1530(2)	5879(2)	13049(2)	19(1)
O(7)	5041(2)	7470(2)	7195(2)	20(1)
O(8)	2128(3)	2801(2)	9197(2)	22(1)
O(9)	814(2)	3354(2)	12323(2)	22(1)
O(10)	2205(2)	10128(2)	11920(2)	25(1)
C(1)	5853(3)	2595(3)	1858(3)	17(1)
C(2)	6734(3)	3149(3)	404(3)	16(1)
C(3)	7024(3)	2299(3)	-927(3)	17(1)
C(4)	7896(3)	3199(3)	-2041(3)	18(1)
C(5)	9169(3)	3824(3)	-4219(3)	22(1)
C(6)	9308(3)	5182(3)	-3372(3)	20(1)
C(7)	8477(3)	4830(3)	-1917(3)	18(1)
C(8)	8134(3)	5659(3)	-540(3)	16(1)
C(9)	7252(3)	4768(3)	597(3)	16(1)
C(10)	6722(3)	5306(3)	2113(3)	17(1)
C(11)	6787(3)	1147(3)	2625(3)	18(1)
C(12)	5866(3)	654(3)	4097(3)	20(1)
C(13)	9313(3)	8259(3)	-1326(3)	21(1)
C(14)	2436(3)	7308(3)	12446(3)	16(1)
C(15)	2980(3)	6806(3)	10862(3)	17(1)
C(16)	3917(3)	7669(3)	9780(3)	18(1)
C(17)	4173(3)	6824(3)	8427(3)	17(1)
C(18)	5011(3)	6294(3)	6079(3)	21(1)
C(19)	4171(3)	4944(3)	6532(3)	19(1)
C(20)	3595(3)	5227(3)	8093(3)	18(1)
C(21)	2706(3)	4355(3)	9248(3)	16(1)
C(22)	2404(3)	5205(3)	10618(3)	16(1)
C(23)	1506(3)	4646(3)	12004(3)	19(1)

C(24)	1228(3)	8834(3)	12579(3)	18(1)
C(25)	681(4)	9177(3)	14220(3)	24(1)
C(26)	2565(4)	1792(3)	7903(3)	23(1)

Table S5. Bond lengths [Å] and angles [°] for i19180.

O(1)-C(10)	1.365(3)
O(1)-C(1)	1.460(3)
O(2)-C(4)	1.369(3)
O(2)-C(5)	1.376(3)
O(3)-C(8)	1.349(3)
O(3)-C(13)	1.432(3)
O(4)-C(10)	1.218(3)
O(5)-C(11)	1.421(3)
O(6)-C(23)	1.363(3)
O(6)-C(14)	1.456(3)
O(7)-C(17)	1.369(3)
O(7)-C(18)	1.375(3)
O(8)-C(21)	1.345(3)
O(8)-C(26)	1.433(3)
O(9)-C(23)	1.214(3)
O(10)-C(24)	1.418(3)
C(1)-C(2)	1.507(3)
C(1)-C(11)	1.531(3)
C(2)-C(3)	1.375(4)
C(2)-C(9)	1.392(3)
C(3)-C(4)	1.389(3)
C(4)-C(7)	1.410(4)
C(5)-C(6)	1.340(4)
C(6)-C(7)	1.450(4)
C(7)-C(8)	1.407(3)
C(8)-C(9)	1.403(3)
C(9)-C(10)	1.453(3)
C(11)-C(12)	1.520(3)
C(14)-C(15)	1.503(3)
C(14)-C(24)	1.529(3)
C(15)-C(16)	1.379(3)
C(15)-C(22)	1.400(4)

C(16)-C(17)	1.387(4)
C(17)-C(20)	1.412(4)
C(18)-C(19)	1.338(4)
C(19)-C(20)	1.454(4)
C(20)-C(21)	1.409(3)
C(21)-C(22)	1.406(4)
C(22)-C(23)	1.463(3)
C(24)-C(25)	1.523(3)
C(10)-O(1)-C(1)	110.52(18)
C(4)-O(2)-C(5)	106.33(19)
C(8)-O(3)-C(13)	119.57(19)
C(23)-O(6)-C(14)	111.05(18)
C(17)-O(7)-C(18)	106.29(19)
C(21)-O(8)-C(26)	119.4(2)
O(1)-C(1)-C(2)	104.04(19)
O(1)-C(1)-C(11)	108.63(19)
C(2)-C(1)-C(11)	113.9(2)
C(3)-C(2)-C(9)	122.7(2)
C(3)-C(2)-C(1)	129.4(2)
C(9)-C(2)-C(1)	107.9(2)
C(2)-C(3)-C(4)	113.8(2)
O(2)-C(4)-C(3)	123.4(2)
O(2)-C(4)-C(7)	110.0(2)
C(3)-C(4)-C(7)	126.6(2)
C(6)-C(5)-O(2)	112.1(2)
C(5)-C(6)-C(7)	106.7(2)
C(8)-C(7)-C(4)	117.7(2)
C(8)-C(7)-C(6)	137.5(3)
C(4)-C(7)-C(6)	104.8(2)
O(3)-C(8)-C(9)	116.1(2)
O(3)-C(8)-C(7)	127.4(2)
C(9)-C(8)-C(7)	116.5(2)
C(2)-C(9)-C(8)	122.8(2)
C(2)-C(9)-C(10)	108.7(2)
C(8)-C(9)-C(10)	128.5(2)
O(4)-C(10)-O(1)	119.6(2)
O(4)-C(10)-C(9)	131.7(2)

O(1)-C(10)-C(9)	108.8(2)
O(5)-C(11)-C(12)	112.8(2)
O(5)-C(11)-C(1)	104.01(19)
C(12)-C(11)-C(1)	111.9(2)
O(6)-C(14)-C(15)	104.04(19)
O(6)-C(14)-C(24)	108.79(19)
C(15)-C(14)-C(24)	115.3(2)
C(16)-C(15)-C(22)	122.5(2)
C(16)-C(15)-C(14)	129.3(2)
C(22)-C(15)-C(14)	108.2(2)
C(15)-C(16)-C(17)	113.6(2)
O(7)-C(17)-C(16)	123.1(2)
O(7)-C(17)-C(20)	110.1(2)
C(16)-C(17)-C(20)	126.8(2)
C(19)-C(18)-O(7)	112.3(2)
C(18)-C(19)-C(20)	106.7(2)
C(21)-C(20)-C(17)	118.0(2)
C(21)-C(20)-C(19)	137.4(3)
C(17)-C(20)-C(19)	104.6(2)
O(8)-C(21)-C(22)	116.3(2)
O(8)-C(21)-C(20)	127.7(2)
C(22)-C(21)-C(20)	116.0(2)
C(15)-C(22)-C(21)	123.0(2)
C(15)-C(22)-C(23)	108.1(2)
C(21)-C(22)-C(23)	128.9(2)
O(9)-C(23)-O(6)	119.8(2)
O(9)-C(23)-C(22)	131.6(2)
O(6)-C(23)-C(22)	108.6(2)
O(10)-C(24)-C(25)	112.5(2)
O(10)-C(24)-C(14)	104.22(18)
C(25)-C(24)-C(14)	111.0(2)

Symmetry transformations used to generate equivalent atoms:

Table S6. Anisotropic displacement parameters ($Å^2 x 10^3$)for i19180. The anisotropic displacement factor exponent takes the form: $-2\pi^2[h^2a^{*2}U^{11} + ... + 2 h k a^* b^* U^{12}]$

	U^{11}	U^{22}	U^{33}	U^{23}	U^{13}	U^{12}
O(1)	27(1)	12(1)	15(1)	-1(1)	4(1)	-2(1)
O(2)	29(1)	21(1)	15(1)	-5(1)	3(1)	0(1)
O(3)	29(1)	14(1)	17(1)	0(1)	4(1)	-5(1)
O(4)	30(1)	14(1)	16(1)	0(1)	3(1)	-2(1)
O(5)	41(1)	11(1)	22(1)	0(1)	2(1)	2(1)
O(6)	26(1)	14(1)	17(1)	0(1)	5(1)	0(1)
O(7)	25(1)	20(1)	17(1)	3(1)	3(1)	-3(1)
O(8)	31(1)	14(1)	22(1)	-4(1)	6(1)	-4(1)
O(9)	29(1)	14(1)	23(1)	0(1)	8(1)	-1(1)
O(10)	27(1)	14(1)	34(1)	2(1)	7(1)	2(1)
C(1)	25(1)	11(1)	16(1)	-2(1)	2(1)	2(1)
C(2)	16(1)	16(1)	18(1)	2(1)	-2(1)	2(1)
C(3)	20(1)	12(1)	18(1)	-1(1)	-1(1)	0(1)
C(4)	21(1)	18(1)	15(1)	-2(1)	-1(1)	3(1)
C(5)	26(1)	25(1)	15(1)	1(1)	4(1)	1(1)
C(6)	20(1)	21(1)	17(1)	3(1)	1(1)	-1(1)
C(7)	20(1)	16(1)	17(1)	1(1)	-1(1)	3(1)
C(8)	20(1)	14(1)	14(1)	0(1)	0(1)	2(1)
C(9)	18(1)	14(1)	17(1)	0(1)	-1(1)	2(1)
C(10)	21(1)	13(1)	16(1)	2(1)	1(1)	2(1)
C(11)	22(1)	14(1)	18(1)	1(1)	-2(1)	-1(1)
C(12)	27(1)	16(1)	18(1)	3(1)	-1(1)	-3(1)
C(13)	25(1)	15(1)	22(1)	3(1)	4(1)	-3(1)
C(14)	19(1)	14(1)	16(1)	1(1)	2(1)	0(1)
C(15)	16(1)	16(1)	18(1)	-1(1)	-2(1)	4(1)
C(16)	21(1)	14(1)	18(1)	0(1)	-1(1)	-1(1)
C(17)	18(1)	17(1)	17(1)	3(1)	1(1)	2(1)
C(18)	24(1)	24(1)	13(1)	0(1)	2(1)	2(1)
C(19)	22(1)	21(1)	14(1)	-3(1)	-1(1)	2(1)
C(20)	20(1)	16(1)	17(1)	-1(1)	-2(1)	2(1)
C(21)	18(1)	14(1)	17(1)	-1(1)	0(1)	2(1)
C(22)	16(1)	13(1)	19(1)	1(1)	-1(1)	1(1)
C(23)	22(1)	15(1)	20(1)	1(1)	2(1)	5(1)

C(24)	21(1)	15(1)	18(1)	-1(1)	1(1)	1(1)
C(25)	33(2)	19(1)	21(1)	-2(1)	4(1)	6(1)
C(26)	29(1)	17(1)	24(1)	-7(1)	0(1)	-1(1)

X-ray diffraction data of compound (–)-nervione

(Displacement ellipsoids are drawn at the 50% probability level, CCDC-2374994.)

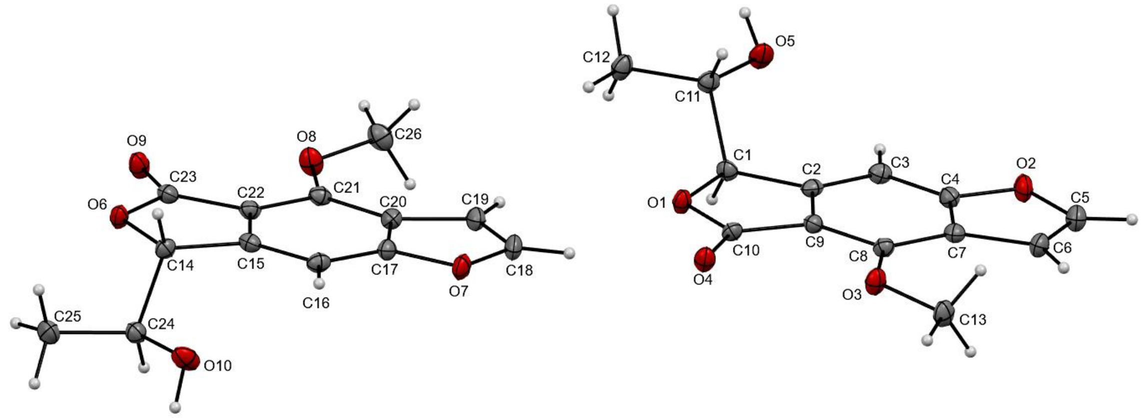

Table S7. Crystal data and structure refinement for i19395.

Identification code	i19395	
Empirical formula	C13 H12 O5	
Formula weight	248.23	
Temperature	100.0(2) K	
Wavelength	1.54178 Å	
Crystal system	Triclinic	
Space group	P 1	
Unit cell dimensions	a = 7.75460(10) Å	α= 89.8420(10)°.
	b = 8.07600(10) Å	β= 87.6090(10)°.
	c = 8.86760(10) Å	γ = 88.4030(10)°.
Volume	554.644(12) Å^3	
Z	2	
Density (calculated)	1.486 Mg/m^3	
Absorption coefficient	0.972 mm^{-1}	
F(000)	260	
Crystal size	0.332 x 0.158 x 0.056 mm^3	
Theta range for data collection	4.992 to 74.461°.	
Index ranges	-9<=h<=9, -10<=k<=10, -10<=l<=11	
Reflections collected	15001	
Independent reflections	4108 [R(int) = 0.0565]	
Completeness to theta = 67.679°	99.3 %	
Absorption correction	Numerical	
Max. and min. transmission	1 and 0.7569	

Refinement method	Full-matrix least-squares on F^2
Data / restraints / parameters	4108 / 5 / 338
Goodness-of-fit on F^2	1.097
Final R indices [I>2sigma(I)]	R1 = 0.0313, wR2 = 0.0786
R indices (all data)	R1 = 0.0330, wR2 = 0.0790
Absolute structure parameter	0.03(7)
Extinction coefficient	0.021(2)
Largest diff. peak and hole	0.304 and -0.319 e.Å^{-3}

Table S8. Atomic coordinates (x 10^4) and equivalent isotropic displacement parameters (Å^{2}x 10^3) for i19395. U(eq) is defined as one third of the trace of the orthogonalized U^{ij} tensor.

	x	y	z	U(eq)
O(1)	4111(2)	5960(2)	7161(2)	15(1)
O(2)	1671(2)	7420(2)	13444(2)	18(1)
O(3)	1443(2)	2782(2)	10186(2)	16(1)
O(4)	3104(2)	3390(2)	7227(2)	16(1)
O(5)	3152(2)	10138(2)	8468(2)	21(1)
O(6)	8468(2)	4124(2)	-3055(2)	15(1)
O(7)	4951(2)	2533(2)	2800(2)	17(1)
O(8)	7871(2)	7204(2)	802(2)	19(1)
O(9)	9183(2)	6652(2)	-2329(2)	19(1)
O(10)	7789(2)	-126(2)	-1927(2)	21(1)
C(1)	4141(3)	7410(3)	8138(2)	13(1)
C(2)	3263(3)	6856(3)	9591(3)	13(1)
C(3)	2967(3)	7706(3)	10921(3)	14(1)
C(4)	2096(3)	6802(3)	12037(2)	14(1)
C(5)	824(3)	6174(3)	14215(2)	18(1)
C(6)	682(3)	4818(3)	13369(3)	17(1)
C(7)	1522(3)	5174(3)	11910(3)	13(1)
C(8)	1861(3)	4341(3)	10537(3)	12(1)
C(9)	2739(3)	5235(3)	9398(2)	12(1)
C(10)	3273(3)	4698(3)	7883(2)	13(1)
C(11)	3207(3)	8856(3)	7369(2)	14(1)
C(12)	4129(3)	9350(3)	5899(2)	17(1)
C(13)	681(3)	1742(3)	11324(2)	17(1)
C(14)	7558(3)	2699(3)	-2445(3)	13(1)
C(15)	7019(3)	3202(3)	-864(2)	13(1)
C(16)	6081(3)	2331(3)	214(3)	14(1)
C(17)	5822(3)	3180(3)	1569(3)	13(1)
C(18)	4984(3)	3713(3)	3917(2)	18(1)
C(19)	5827(3)	5064(3)	3465(3)	16(1)
C(20)	6399(3)	4775(3)	1902(2)	14(1)
C(21)	7292(3)	5649(3)	747(3)	13(1)
C(22)	7596(3)	4804(3)	-621(3)	13(1)
C(23)	8488(3)	5356(3)	-2007(3)	15(1)

C(24)	8770(3)	1171(3)	-2583(2)	14(1)
C(25)	9317(3)	826(3)	-4222(3)	21(1)
C(26)	7430(3)	8209(3)	2097(3)	20(1)

Table S9. Bond lengths [Å] and angles [°] for i19395.

O(1)-C(10)	1.368(2)
O(1)-C(1)	1.460(2)
O(2)-C(4)	1.368(3)
O(2)-C(5)	1.381(3)
O(3)-C(8)	1.349(3)
O(3)-C(13)	1.431(3)
O(4)-C(10)	1.220(3)
O(5)-C(11)	1.423(2)
O(6)-C(23)	1.364(3)
O(6)-C(14)	1.457(2)
O(7)-C(17)	1.370(3)
O(7)-C(18)	1.378(3)
O(8)-C(21)	1.347(3)
O(8)-C(26)	1.432(3)
O(9)-C(23)	1.218(3)
O(10)-C(24)	1.421(2)
C(1)-C(2)	1.505(3)
C(1)-C(11)	1.531(3)
C(2)-C(3)	1.374(3)
C(2)-C(9)	1.395(3)
C(3)-C(4)	1.391(3)
C(4)-C(7)	1.407(3)
C(5)-C(6)	1.339(3)
C(6)-C(7)	1.455(3)
C(7)-C(8)	1.405(3)
C(8)-C(9)	1.403(3)
C(9)-C(10)	1.453(3)
C(11)-C(12)	1.518(3)
C(14)-C(15)	1.500(3)
C(14)-C(24)	1.532(3)
C(15)-C(16)	1.380(3)
C(15)-C(22)	1.401(3)

C(16)-C(17)	1.389(3)
C(17)-C(20)	1.412(3)
C(18)-C(19)	1.339(3)
C(19)-C(20)	1.455(3)
C(20)-C(21)	1.411(3)
C(21)-C(22)	1.402(3)
C(22)-C(23)	1.460(3)
C(24)-C(25)	1.521(3)
C(10)-O(1)-C(1)	110.50(16)
C(4)-O(2)-C(5)	106.03(17)
C(8)-O(3)-C(13)	119.62(17)
C(23)-O(6)-C(14)	110.71(16)
C(17)-O(7)-C(18)	106.08(16)
C(21)-O(8)-C(26)	119.29(18)
O(1)-C(1)-C(2)	104.07(16)
O(1)-C(1)-C(11)	108.55(16)
C(2)-C(1)-C(11)	113.96(17)
C(3)-C(2)-C(9)	122.5(2)
C(3)-C(2)-C(1)	129.46(19)
C(9)-C(2)-C(1)	108.06(19)
C(2)-C(3)-C(4)	113.75(19)
O(2)-C(4)-C(3)	123.1(2)
O(2)-C(4)-C(7)	110.35(19)
C(3)-C(4)-C(7)	126.5(2)
C(6)-C(5)-O(2)	112.31(19)
C(5)-C(6)-C(7)	106.5(2)
C(8)-C(7)-C(4)	118.0(2)
C(8)-C(7)-C(6)	137.1(2)
C(4)-C(7)-C(6)	104.9(2)
O(3)-C(8)-C(9)	116.25(19)
O(3)-C(8)-C(7)	127.6(2)
C(9)-C(8)-C(7)	116.19(19)
C(2)-C(9)-C(8)	123.0(2)
C(2)-C(9)-C(10)	108.51(19)
C(8)-C(9)-C(10)	128.47(19)
O(4)-C(10)-O(1)	119.6(2)
O(4)-C(10)-C(9)	131.59(19)

O(1)-C(10)-C(9)	108.82(17)
O(5)-C(11)-C(12)	112.88(17)
O(5)-C(11)-C(1)	103.96(16)
C(12)-C(11)-C(1)	111.89(18)
O(6)-C(14)-C(15)	104.19(17)
O(6)-C(14)-C(24)	108.53(16)
C(15)-C(14)-C(24)	115.46(17)
C(16)-C(15)-C(22)	122.9(2)
C(16)-C(15)-C(14)	128.8(2)
C(22)-C(15)-C(14)	108.26(19)
C(15)-C(16)-C(17)	113.26(19)
O(7)-C(17)-C(16)	122.99(19)
O(7)-C(17)-C(20)	110.19(19)
C(16)-C(17)-C(20)	126.8(2)
C(19)-C(18)-O(7)	112.48(19)
C(18)-C(19)-C(20)	106.4(2)
C(21)-C(20)-C(17)	118.0(2)
C(21)-C(20)-C(19)	137.2(2)
C(17)-C(20)-C(19)	104.8(2)
O(8)-C(21)-C(22)	116.3(2)
O(8)-C(21)-C(20)	127.6(2)
C(22)-C(21)-C(20)	116.07(19)
C(15)-C(22)-C(21)	122.8(2)
C(15)-C(22)-C(23)	107.99(19)
C(21)-C(22)-C(23)	129.2(2)
O(9)-C(23)-O(6)	119.7(2)
O(9)-C(23)-C(22)	131.5(2)
O(6)-C(23)-C(22)	108.85(18)
O(10)-C(24)-C(25)	112.37(18)
O(10)-C(24)-C(14)	104.10(16)
C(25)-C(24)-C(14)	111.20(18)

Symmetry transformations used to generate equivalent atoms:

Table S10. Anisotropic displacement parameters ($Å^2$x 10^3)for i19395. The anisotropic displacement factor exponent takes the form: $-2\pi^2[h^2a^{*2}U^{11} + ... + 2 h k a^* b^* U^{12}]$

	U^{11}	U^{22}	U^{33}	U^{23}	U^{13}	U^{12}
O(1)	22(1)	10(1)	12(1)	1(1)	4(1)	-2(1)
O(2)	23(1)	18(1)	11(1)	-3(1)	4(1)	-1(1)
O(3)	24(1)	11(1)	14(1)	0(1)	3(1)	-5(1)
O(4)	25(1)	11(1)	14(1)	-1(1)	3(1)	-1(1)
O(5)	35(1)	9(1)	17(1)	1(1)	3(1)	1(1)
O(6)	22(1)	12(1)	12(1)	2(1)	6(1)	0(1)
O(7)	20(1)	18(1)	12(1)	4(1)	4(1)	-2(1)
O(8)	25(1)	12(1)	21(1)	-3(1)	6(1)	-4(1)
O(9)	23(1)	12(1)	20(1)	1(1)	9(1)	-1(1)
O(10)	21(1)	10(1)	31(1)	3(1)	7(1)	1(1)
C(1)	15(1)	10(1)	14(1)	-1(1)	1(1)	0(1)
C(2)	10(1)	12(1)	16(1)	3(1)	-2(1)	1(1)
C(3)	13(1)	12(1)	16(1)	0(1)	-1(1)	-1(1)
C(4)	14(1)	14(1)	13(1)	-1(1)	-1(1)	2(1)
C(5)	20(1)	22(1)	13(1)	1(1)	2(1)	1(1)
C(6)	17(1)	18(1)	14(1)	4(1)	2(1)	0(1)
C(7)	12(1)	14(1)	13(1)	1(1)	-1(1)	2(1)
C(8)	14(1)	10(1)	13(1)	1(1)	-1(1)	0(1)
C(9)	12(1)	11(1)	14(1)	1(1)	1(1)	1(1)
C(10)	15(1)	10(1)	14(1)	3(1)	1(1)	1(1)
C(11)	17(1)	11(1)	14(1)	1(1)	0(1)	-1(1)
C(12)	23(1)	14(1)	14(1)	3(1)	1(1)	-3(1)
C(13)	21(1)	14(1)	17(1)	3(1)	5(1)	-4(1)
C(14)	15(1)	11(1)	14(1)	2(1)	1(1)	-1(1)
C(15)	11(1)	12(1)	15(1)	-1(1)	-1(1)	2(1)
C(16)	15(1)	11(1)	16(1)	2(1)	-1(1)	-1(1)
C(17)	12(1)	15(1)	13(1)	3(1)	2(1)	0(1)
C(18)	19(1)	24(1)	10(1)	1(1)	3(1)	1(1)
C(19)	18(1)	20(1)	11(1)	-2(1)	0(1)	3(1)
C(20)	14(1)	14(1)	12(1)	1(1)	-1(1)	1(1)
C(21)	12(1)	11(1)	16(1)	1(1)	0(1)	0(1)
C(22)	12(1)	11(1)	16(1)	2(1)	1(1)	1(1)
C(23)	13(1)	14(1)	17(1)	2(1)	2(1)	4(1)

C(24)	15(1)	13(1)	15(1)	0(1)	2(1)	0(1)
C(25)	26(1)	18(1)	16(1)	-1(1)	4(1)	6(1)
C(26)	22(1)	16(1)	21(1)	-6(1)	1(1)	-1(1)

6. Reference

(1) (a) Thomsen, I.; TORSSELL, K. G. Iodination of resorcinol, 5-methoxyresorcinol, phloroglucinol and resorcyclic acid. *Acta Chem. Scand. (Copenhagen. 1989)* **1991**, *45* (5), 539-542. (b) Almetwali, F.; Rouden, J.; Blanchet, J. A Strategy for Improving the Efficiency of Boronic Acid Catalysis in the Synthesis of Amides. *Eur. J. Org. Chem.* **2023**, *26* (45), e202300720.

(2) (a) Slutskyy, Y.; Jewell, W. T.; Lucero, C. G. Syntheses of (−)-Tatarinoid A,(±)-Tatarinoid B, and (−)-Tatarinoid C. *Tetrahedron Lett.* **2013**, *54* (3), 210-212. (b) Christy, M. P.; Johnson, T.; McNerlin, C. D.; Woodard, J.; Nelson, A. T.; Lim, B.; Hamilton, T. L.; Freiberg, K. M.; Siegel, D. Total synthesis of micrococcin P1 through scalable thiazole forming reactions of cysteine derivatives and nitriles. *Org. Lett.* **2020**, *22* (6), 2365-2370.

(3) (a) Shiina, I.; Takasuna, Y.-j.; Suzuki, R.-s.; Oshiumi, H.; Komiyama, Y.; Hitomi, S.; Fukui, H. Stereoselective total synthesis of the proposed structure of 2-epibotcinolide. *Org. Lett.* **2006**, *8* (23), 5279-5282. (b) Mandal, S.; Mahananda, D.; Paladugu, D.; Thirupathi, B. Total Synthesis and Determination of the Absolute Configuration of Berkeleylactone I. *J. Org. Chem.* **2024**, *89* (6), 4165-4175.

(4) Nguyen, T.-H.; Castanet, A.-S.; Mortier, J. Directed ortho-metalation of unprotected benzoic acids. Methodology and regioselective synthesis of useful contiguously 3-and 6-substituted 2-methoxybenzoic acid building blocks. *Org. Lett.* **2006**, *8* (4), 765-768.
